# Cinacalcet versus Placebo for secondary hyperparathyroidism in chronic kidney disease patients: a meta-analysis of randomized controlled trials and trial sequential analysis

**DOI:** 10.1038/s41598-018-21397-8

**Published:** 2018-02-15

**Authors:** Guoqi Wang, Hongyan Liu, Chengzhi Wang, Xiaojian Ji, Weijun Gu, Yiming Mu

**Affiliations:** 1 Department of Orthopedics, Chinese PLA General Hospital, No. 28 Fuxing Road, Haidian District Beijing, 100853 P.R. China; 2 Department of Endocrinology, Chinese PLA General Hospital, No. 28 Fuxing Road, Haidian District Beijing, 100853 P.R. China; 3 Department of rheumatology, Chinese PLA General Hospital, No. 28 Fuxing Road, Haidian District Beijing, 100853 P.R. China

## Abstract

To assess the efficacy and safety of cinacalcet on secondary hyperparathyroidism in patients with chronic kidney disease, Pubmed, Embase, and the Cochrane Central Register of Controlled Trials were searched until March 2016. Trial sequential analysis (TSA) was conducted to control the risks of type I and II errors and calculate required information size (RIS). A total of 25 articles with 8481 participants were included. Compared with controls, cinacalcet administration did not reduce all-cause mortality (RR = 0.97, 95% CI = 0.89–1.05, P = 0.41, TSA-adjusted 95% CI = 0.86–1.08, RIS = 5260, n = 8386) or cardiovascular mortality (RR = 0.95, 95% CI = 0.83–1.07, P = 0.39, TSA-adjusted 95% CI = 0.70–1.26, RIS = 3780 n = 5418), but it reduced the incidence of parathyroidectomy (RR = 0.48, 95% CI = 0.40–0.50, P < 0.001, TSA-adjusted 95% CI = 0.39–0.60, RIS = 5787 n = 5488). Cinacalcet increased the risk of hypocalcemia (RR = 8.48, 95% CI = 6.37–11.29, P < 0.001, TSA-adjusted 95% CI = 5.25–13.70, RIS = 6522, n = 7785), nausea (RR = 2.12, 95% CI = 1.62–2.77, P < 0.001, TSA-adjusted 95% CI = 1.45–3.04, RIS = 4684, n = 7512), vomiting (RR = 2.00, 95% CI = 1.79–2.24, P < 0.001, TSA-adjusted 95% CI = 1.77–2.26, RIS = 1374, n = 7331) and diarrhea (RR = 1.17, 95% CI = 1.05–1.32, P = 0.006, TSA-adjusted 95% CI = 1.02–1.36, RIS = 8388, n = 6116). Cinacalcet did not significantly reduce the incidence of fractures (RR = 0.58, 95% CI = 0.21–1.59, P = 0.29, TSA-adjusted 95% CI = 0.01–35.11, RIS = 76376, n = 4053). Cinacalcet reduced the incidence of parathyroidectomy, however, it did not reduce all-cause and cardiovascular mortality, and increased the risk of adverse events including hypocalcemia and gastrointestinal disorders.

## Introduction

Secondary hyperparathyroidism (SHPT) is a common and serious complication of chronic kidney disease (CKD), particularly of end-stage renal disease, and deteriorates as kidney function declines^1^. It is characterized by persistently increased serum parathyroid hormone (PTH) concentration and is associated with elevated mortality, cardiovascular disease and bone disease^2–5^. The main pathogenesis of CKD-SHPT includes hypocalcemia, hyperphosphatemia, and vitamin D deficiency; additionally, the increased level of fibroblast growth factor 23 also plays an important role^6^. Hypocalcemia is the main factor in CKD-SHPT that reduces the activation of calcium-sensing receptor (CaSR) in the parathyroid gland^7^, which is down-regulated in patients with CKD-SHPT^8^. Hyperphosphatemia and vitamin D deficiency are also important factors that contribute to CKD-SHPT by reducing serum calcium or by directly contributing to PTH synthesis and secretion^7^. Thus, it is likely that CaSR may be a primary regulator of serum PTH levels.

Cinacalcet, a calcimimetic agent, reduces serum PTH levels by allosterically activating CaSR in the parathyroid gland and by inhibiting PTH secretion^9^, providing a new therapeutic option for patients with SHPT. At the same time, cinacalcet does not increase the levels of serum calcium and phosphorus; therefore, it is superior to the traditional drugs, such as vitamin D receptor activator and phosphate binders. Cinacalcet has been used for the treatment of SHPT in CKD patients for over 10 years since the first approval by the Food and Drug Administration and a number of randomized control trials have studied the efficacy and safety of cinacalcet. However, the patient-level outcomes of cinacalcet are still unclear.

Five reviews^10–14^ examining the effects of cinacalcet on SHPT in CKD patients have been conducted, four of which by the same authors. One meta-analysis study did not analyze patient-level outcomes^14^. One review investigated the association of the effect of four drugs (vitamin D compounds, phosphate binders, cinacalcet and bisphosphonates) on the level of biological parameters with mortality^13^. Trial sequential analysis (TSA) was performed in none of them. In view of these shortcomings and the completion of several additional trials^2,15–18^, we performed this meta-analysis to provide further evidence regarding the effects of cinacalcet on clinical outcomes in patients with CKD. In addition, we analyzed the proportion of patients that achieved the calcium-by-phosphorus product target (Ca × P ≤ 5.5 mg^2^/dL^2^, recommended by KDOQI) and examined their bone turnover markers, including osteocalcin, urine N-telopeptide (urine NTx) and bone-specific alkaline phosphatase (BALP). In addition to CKD staging, we also conducted subgroup analysis according to drug administration duration to search for the source of heterogeneity. Furthermore, TSA was performed in our meta-analysis.

## Results

### Study selection

**Figure 1:**
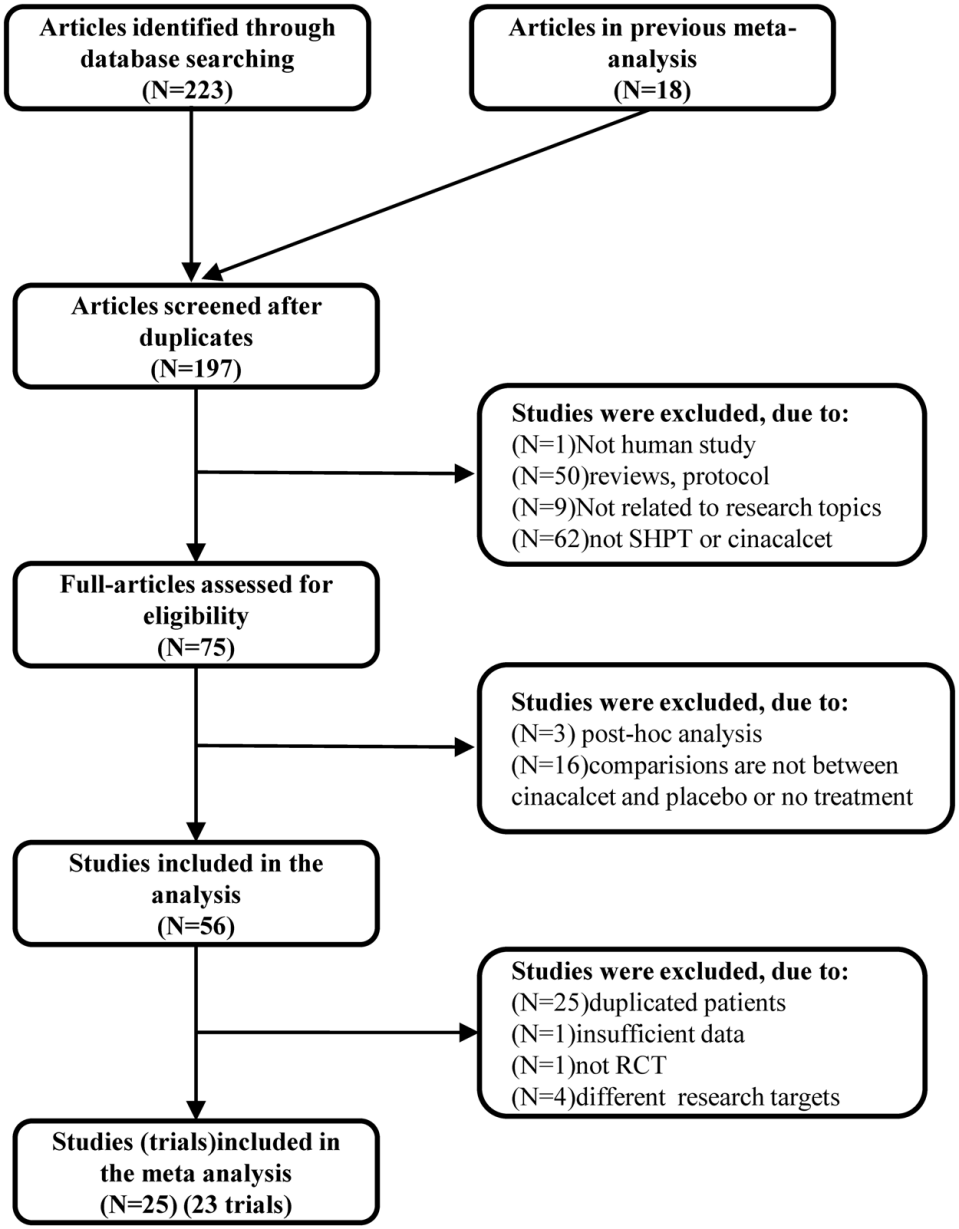
Flow diagram for study selection.

We identified 223 articles initially, of which only 38 studies were regarded as eligibility. Adding to 18 trials with 7446 participants from an earlier meta-analysis up to February 2013^11^, a final total of 25 studies (23 RCTs)^2,3,15–37^ with 8481 participants were included in our paper (Fig. 1).

### Study characteristics

The duration of drug treatment was all within one year except one trial of five years’ duration. Nineteen trials included patients with stage 5 CKD under dialysis treatment. One trial included CKD patients that had received kidney transplants. Two trials included patients with stage 3–4 CKD that had not undergone dialysis or kidney transplantation. The dosage of cinacalcet was between 30 and 180 mg/d in thirteen trials. Placebo was used as control in 16 trials. The use of vitamin D in two groups was not comparable in six trials. More details are shown in Table S1 (Additional file 2).

### Risk of bias and quality of evidence

Figure S1 (Additional file 2) shows the risk of bias in the included trials. The GRADE evidence quality for outcomes is summarized in Table S2 (Additional file 2). The quality of the main outcomes in stage 5 CKD patients under dialysis treatment was moderate to high.

### Clinical outcomes

#### All-cause mortality

**Figure 2:**
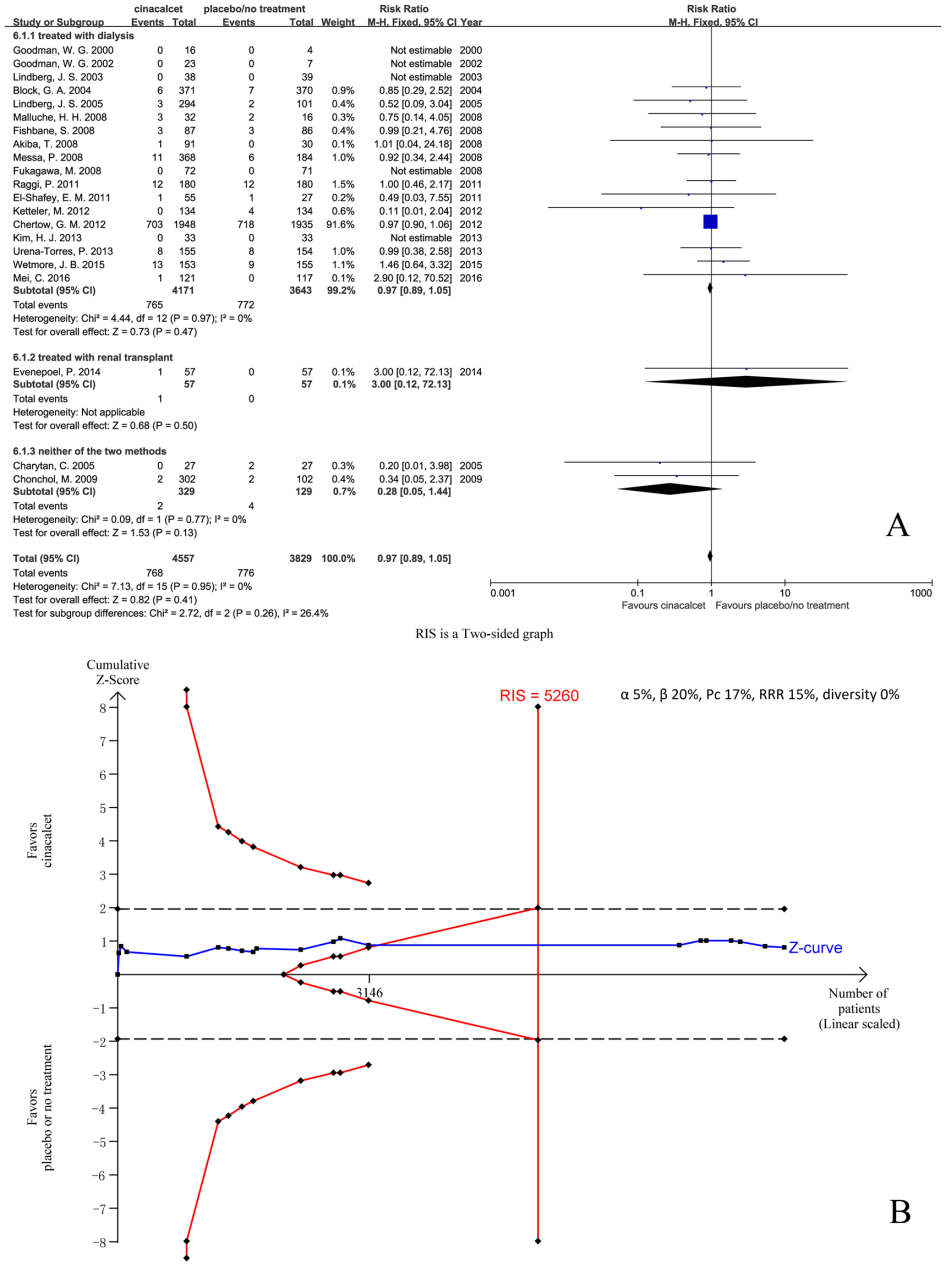
Forest plot (**A**) and trial sequential analysis (**B**) on all-cause mortality in cinacalcet group versus control group. RRR: relative risk reduction, Pc: event proportion in control group.

We included 21 trials with 8386 participants that had reported all-cause mortality in two groups. Our results indicated that cinacalcet did not reduce all-cause mortality compared with placebo or no treatment in patients with SHPT caused by CKD (RR 0.97, 95% CI 0.89 to 1.05, P = 0.41), as determined using a fixed effects model (I^2^ = 0%, P = 0.95) (Fig. 2A).

TSA was conducted based on the 17% all-cause mortality rate in the control group, a 15% relative risk reduction in experimental group, and 0% diversity (D^2^). The required information size (RIS) was 5260 participants. The cumulative Z curve (blue line) crossed neither the trial sequential monitoring boundaries (red inward slash) nor the conventional boundaries (black dotted line); however, it entered the futility area and RIS has been reached (Fig. 2B). The TSA-adjusted 95% CI of RR was 0.86 to 1.08.

#### Cardiovascular mortality

**Figure 3:**
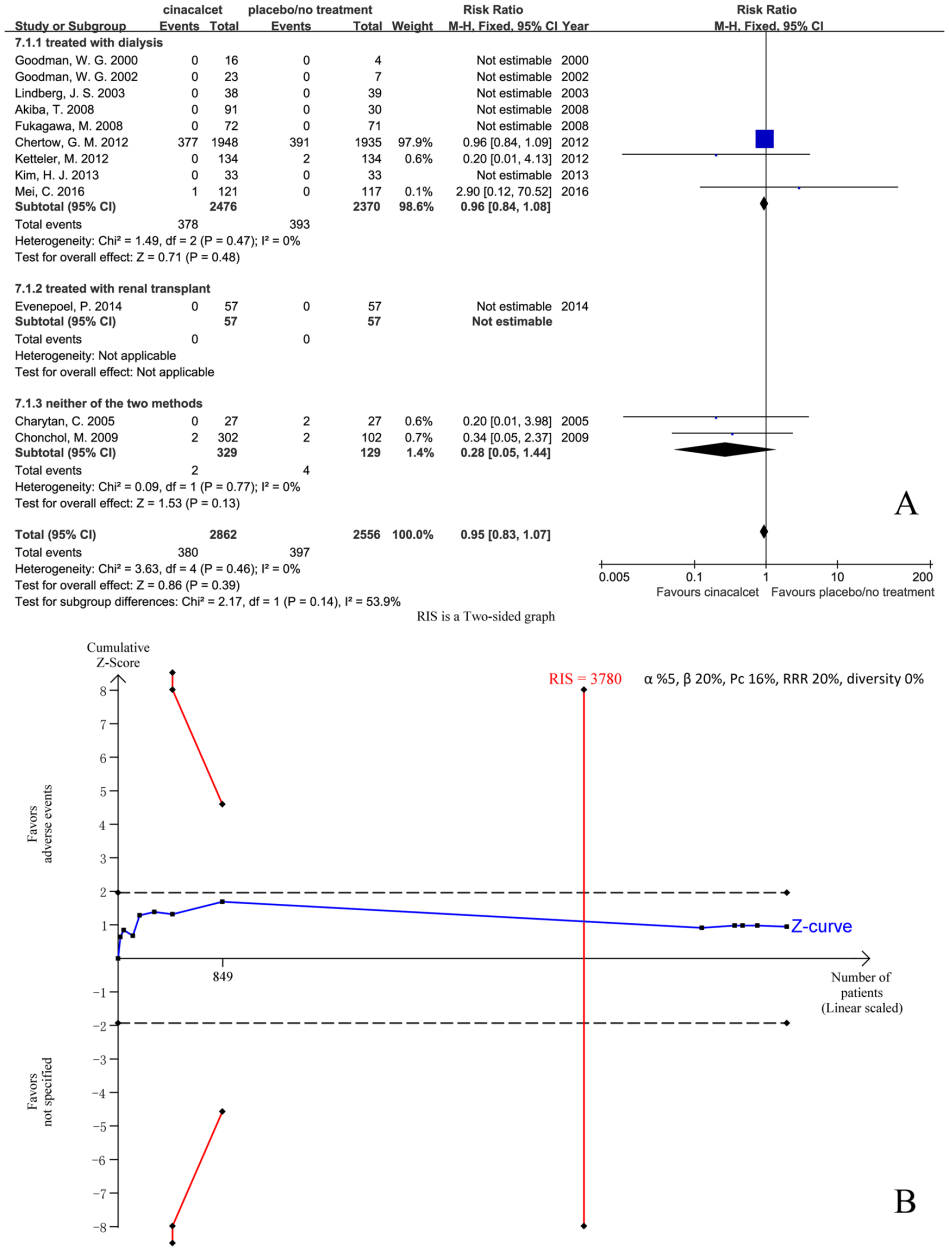
Forest plot (**A**) and trial sequential analysis (**B**) on cardiovascular mortality in cinacalcet group versus control group. RRR: relative risk reduction, Pc: event proportion in control group.

Twelve trials with 5418 participants that had reported cardiovascular mortality were included in our meta-analysis. The results showed that cinacalcet did not reduce cardiovascular mortality compared with placebo or no treatment in patients with SHPT caused by CKD (RR 0.95, 95% CI 0.83 to 1.07, P = 0.39), as determined using a fixed effects model (I^2^ = 0%, P = 0.46) (Fig. 3A).

We conducted TSA on the basis of 16% cardiovascular mortality rate in the control group, a 20% relative risk reduction in the experimental group, and 0% D^2^. RIS was 3780 participants. Although the cumulative Z curve (blue line) did not cross the trial sequential monitoring boundaries (red inward slash) or the conventional boundaries (black dotted line), RIS has been reached (Fig. 3B). The TSA-adjusted 95% CI of RR was 0.70 to 1.26.

#### Parathyroidectomy

**Figure 4:**
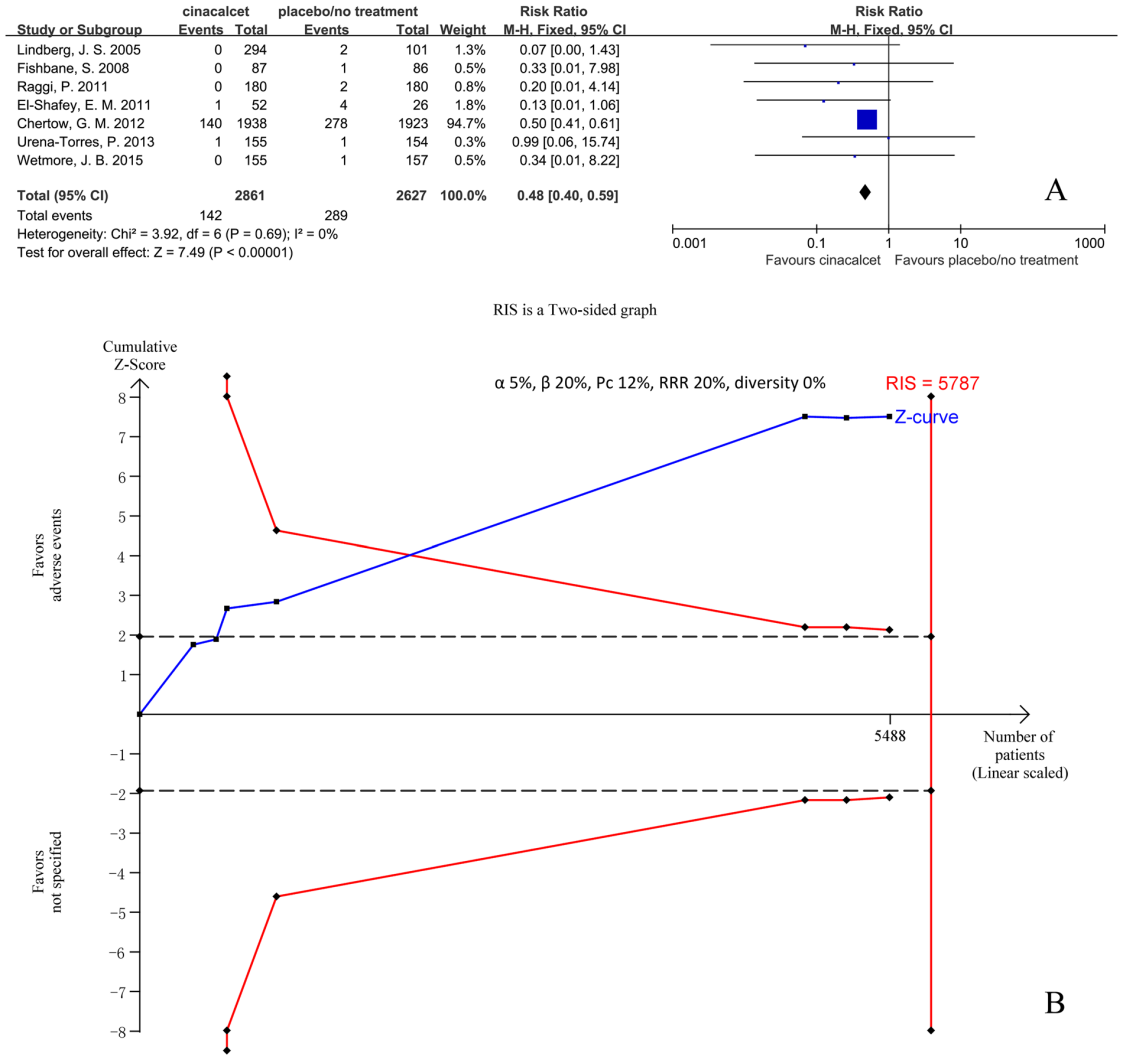
Forest plot (**A**) and trial sequential analysis (**B**) on parathyroidectomy in cinacalcet group versus control group. RRR: relative risk reduction, Pc: event proportion in control group.

Seven trials with 5488 participants that had reported the proportion of patients undergoing parathyroidectomy were included in our meta-analysis. The results showed that cinacalcet reduced the incidence of parathyroidectomy (RR 0.48, 95% CI 0.40 to 0.50, P < 0.001), according to a fixed effects model (I^2^ = 0%, P = 0.69) (Fig. 4A).

TSA was conducted based on the 12% incidence of parathyroidectomy in the control group, a 20% relative risk reduction in the experimental group, and 0% D^2^. RIS was 5787 participants. The cumulative Z curve (blue line) crossed the trial sequential monitoring boundaries (red inward slash) before the RIS has been reached (Fig. 4B). The TSA-adjusted 95% CI of RR was 0.39 to 0.60.

#### Fractures

**Figure 5:**
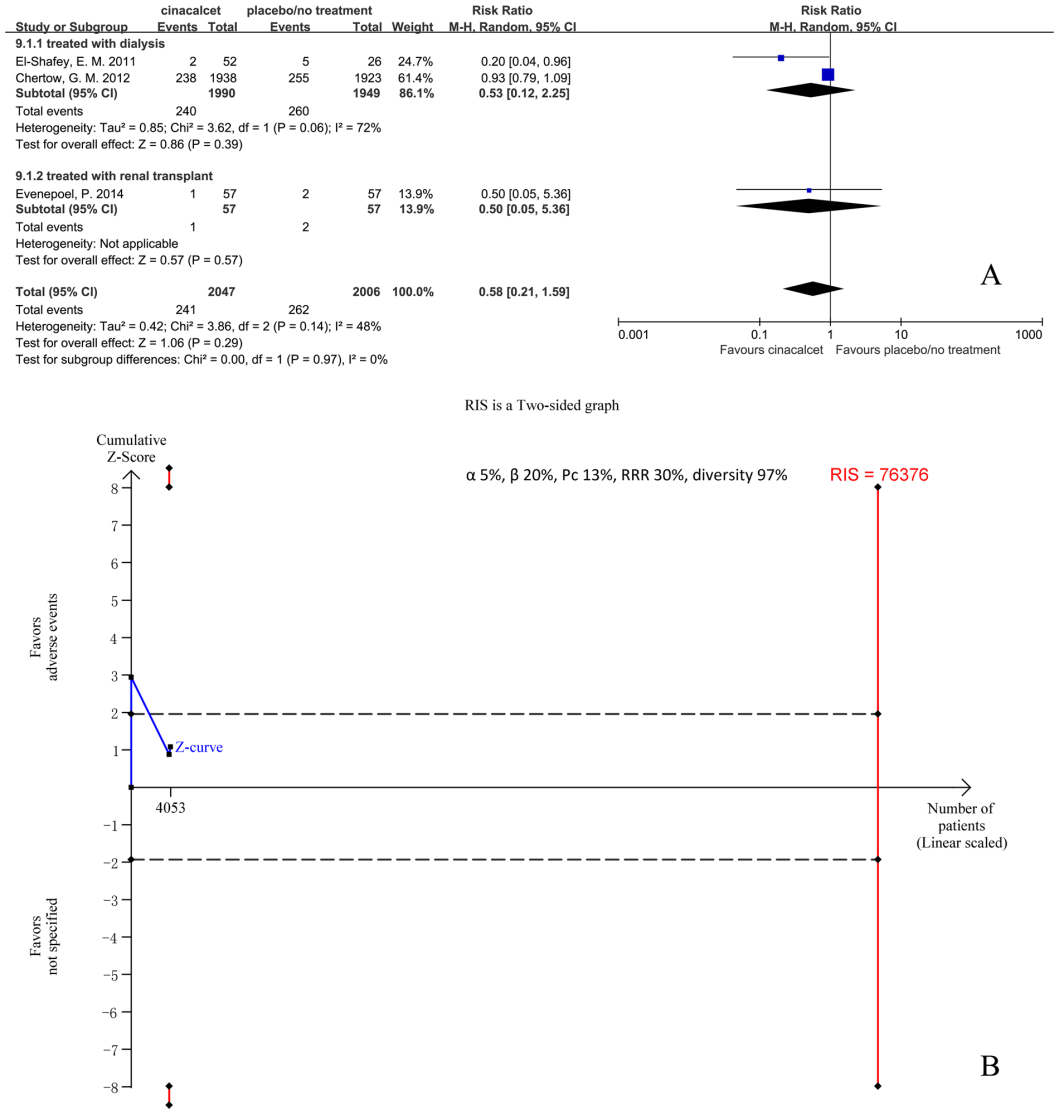
Forest plot (**A**) and trial sequential analysis (**B**) on fractures in cinacalcet group versus control group. RRR: relative risk reduction, Pc: event proportion in control group.

Only three trials with 4053 participants that had reported the incidence of fractures were included in our meta-analysis. The results showed that cinacalcet did not significantly reduced the incidence of fractures (RR 0.58, 95% CI 0.21 to 1.59, P = 0.29), as determined using a random effects model (I^2^ = 72%, P = 0.06) (Fig. 5A).

TSA was conducted based on the 13% incidence of fractures in the control group, a 30% relative risk reduction in the experimental group, and 97% D^2^. RIS was 76376 participants, 5.3% of whom were accrued in our meta-analysis. The cumulative Z curve (blue line) did not cross the conventional boundaries (black dotted line) and RIS was far from reached (Fig. 5B). The TSA-adjusted 95% CI of RR was 0.01 to 35.11.

### Adverse events

#### All adverse events

**Figure 6:**
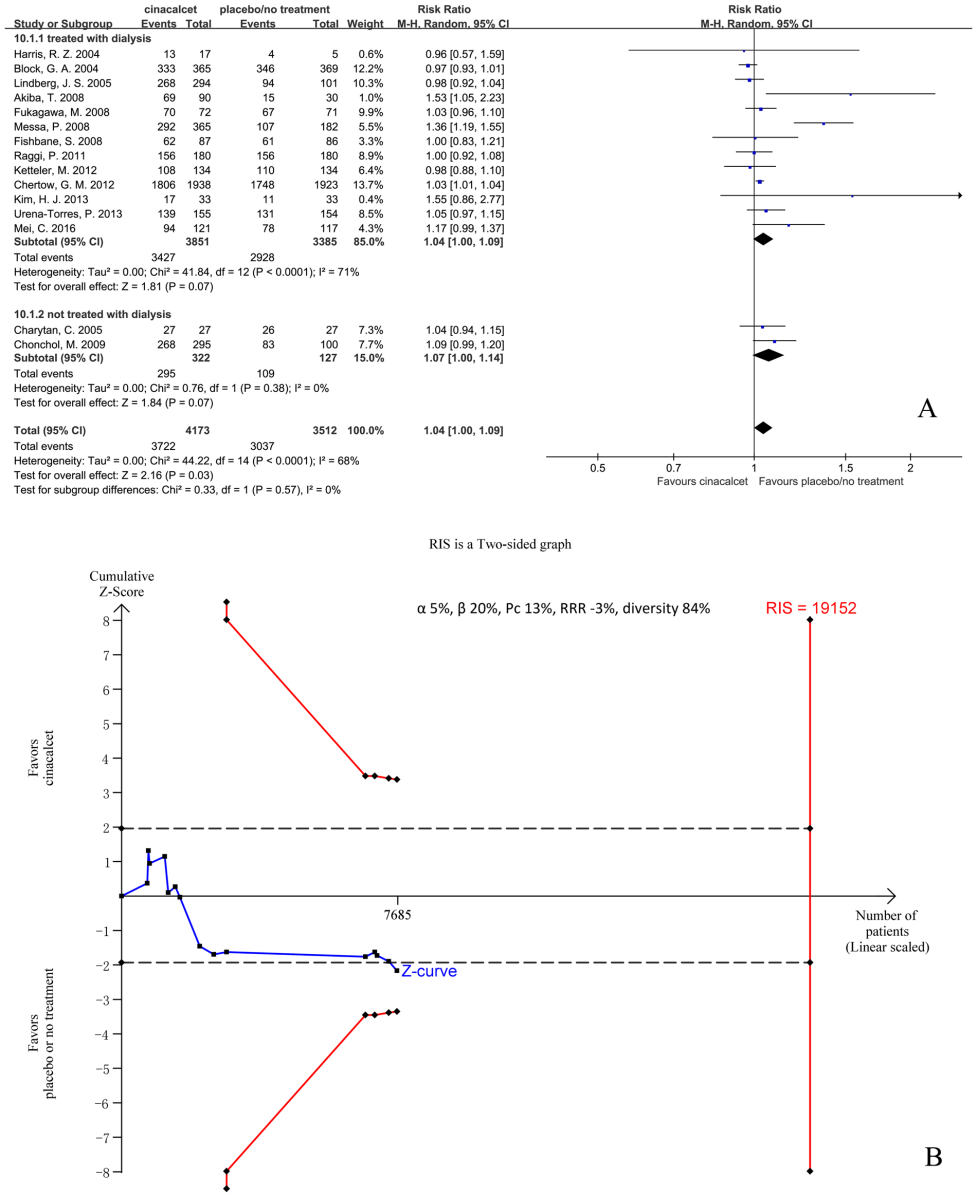
Forest plot (**A**) and trial sequential analysis (**B**) on all adverse events in cinacalcet group versus control group. RRR: relative risk reduction, Pc: event proportion in control group.

Fifteen trials with 7685 participants that had reported the proportion of patients with at least one adverse event during the trials were included in our meta-analysis. The results showed that, compared with placebo or no treatment, cinacalcet increased the number of patients that experienced at least one adverse event (RR 1.04, 95% CI 1.00 to 1.09, P = 0.03), as determined using a random effects model (I^2^ = 68%, P < 0.001) (Fig. 6A).

Subgroup analysis according to CKD staging or drug administration duration, and sensitivity analysis were conducted. Our subgroup analysis showed that drug administration duration, but not CKD staging, influenced the pooled RR (test for subgroup differences: I^2^ = 79%, P = 0.003 vs. I^2^ = 0%, P = 0.57). Our sensitivity analysis indicated that no single study had a significant influence on pooled RR (Figure S2, additional file 2).

TSA was conducted based on the 13% proportion of patients experiencing at least one adverse event in the control group, a 3% relative risk increase in the experimental group, and 84% D^2^. RIS was 19152 participants, 40.1% of whom were accrued in our meta-analysis. The cumulative Z curve (blue line) crossed the conventional boundaries (black dotted line) but did not cross the trial sequential monitoring boundaries (red inward slash), and RIS has not been reached (Fig. 6B). The TSA-adjusted 95% CI of RR was 0.98 to 1.11.

#### Hypocalcemia

**Figure 7:**
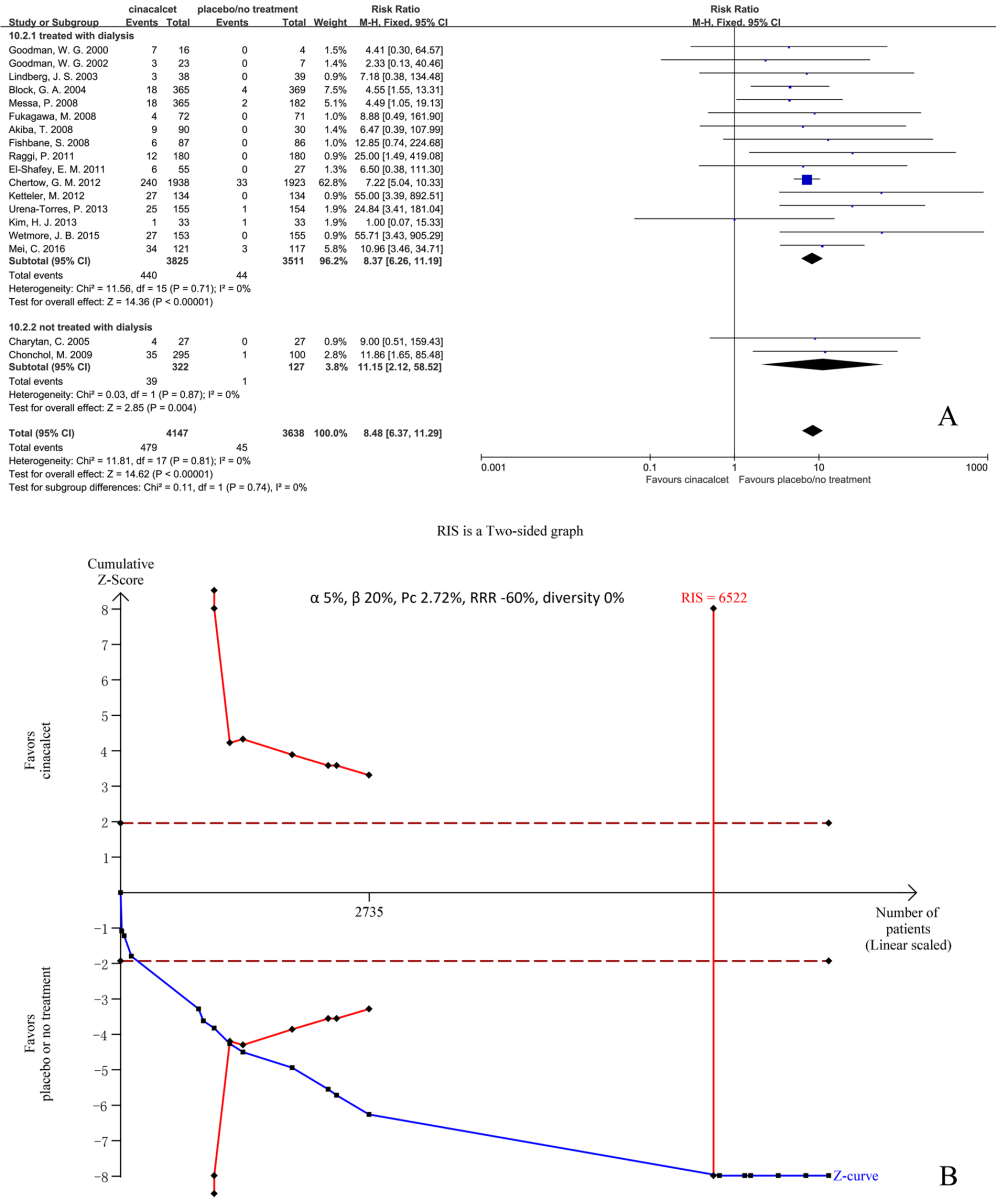
Forest plot (**A**) and trial sequential analysis (**B**) on hypocalcaemia in cinacalcet group versus control group. RRR: relative risk reduction, Pc: event proportion in control group.

Eighteen trials with 7785 participants that had reported the incidence of hypocalcemia were included in our meta-analysis. The results showed that cinacalcet increased the risk of hypocalcemia (RR 8.48, 95% CI 6.37 to 11.29, P < 0.001) as determined using a fixed effects model (I^2^ = 0%, P = 0.81) (Fig. 7A).

RIS was 6522 participants. The cumulative Z curve (blue line) crossed both the conventional boundaries (black dotted line) and the trial sequential monitoring boundaries (red inward slash), and RIS has been reached (Fig. 7B). The TSA-adjusted 95% CI of RR was 5.25 to 13.70.

#### Hypercalcemia

**Figure 8:**
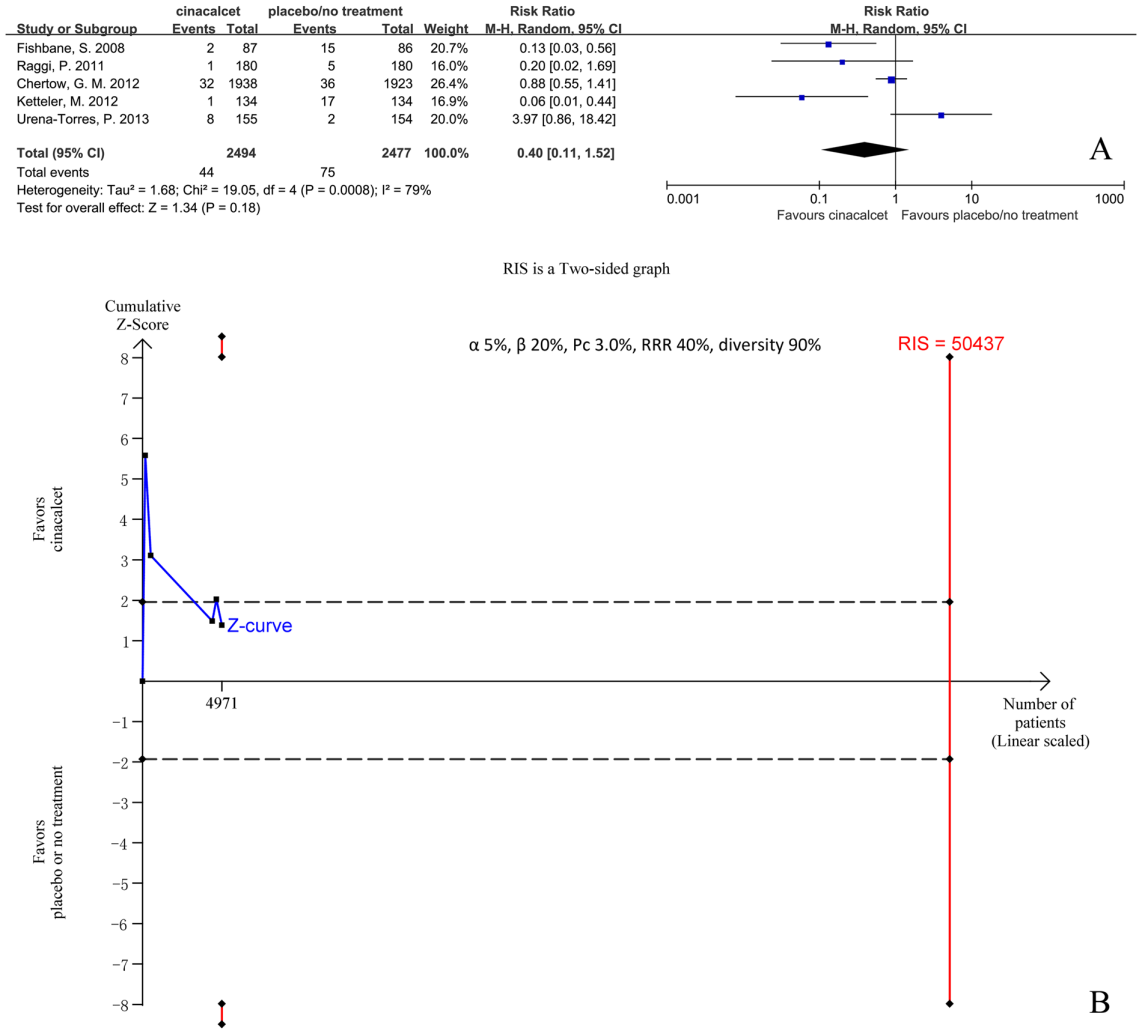
Forest plot (**A**) and trial sequential analysis (**B**) on hypercalcaemia in cinacalcet group versus control group. RRR: relative risk reduction, Pc: event proportion in control group.

Five trials with 4971 participants that had reported the incidence of hypercalcemia were included in our meta-analysis. The results showed that it was uncertain whether cinacalcet reduced the risk of hypercalcemia (RR 0.40, 95% CI 0.11 to 1.52, P = 0.03), as determined using a random effects model (I^2^ = 79%, P < 0.001) (Fig. 8A). Our sensitivity analysis revealed that no single study had a significant influence on pooled RR (Additional file 2: Figure S3).

RIS was 50437 participants, 9.8% of whom were accrued in our meta-analysis. The cumulative Z curve (blue line) did not cross the conventional boundaries (black dotted line) and RIS was far from reached (Fig. 8B). The TSA-adjusted 95% CI of RR was 0.00 to 80.89.

#### Nausea

**Figure 9:**
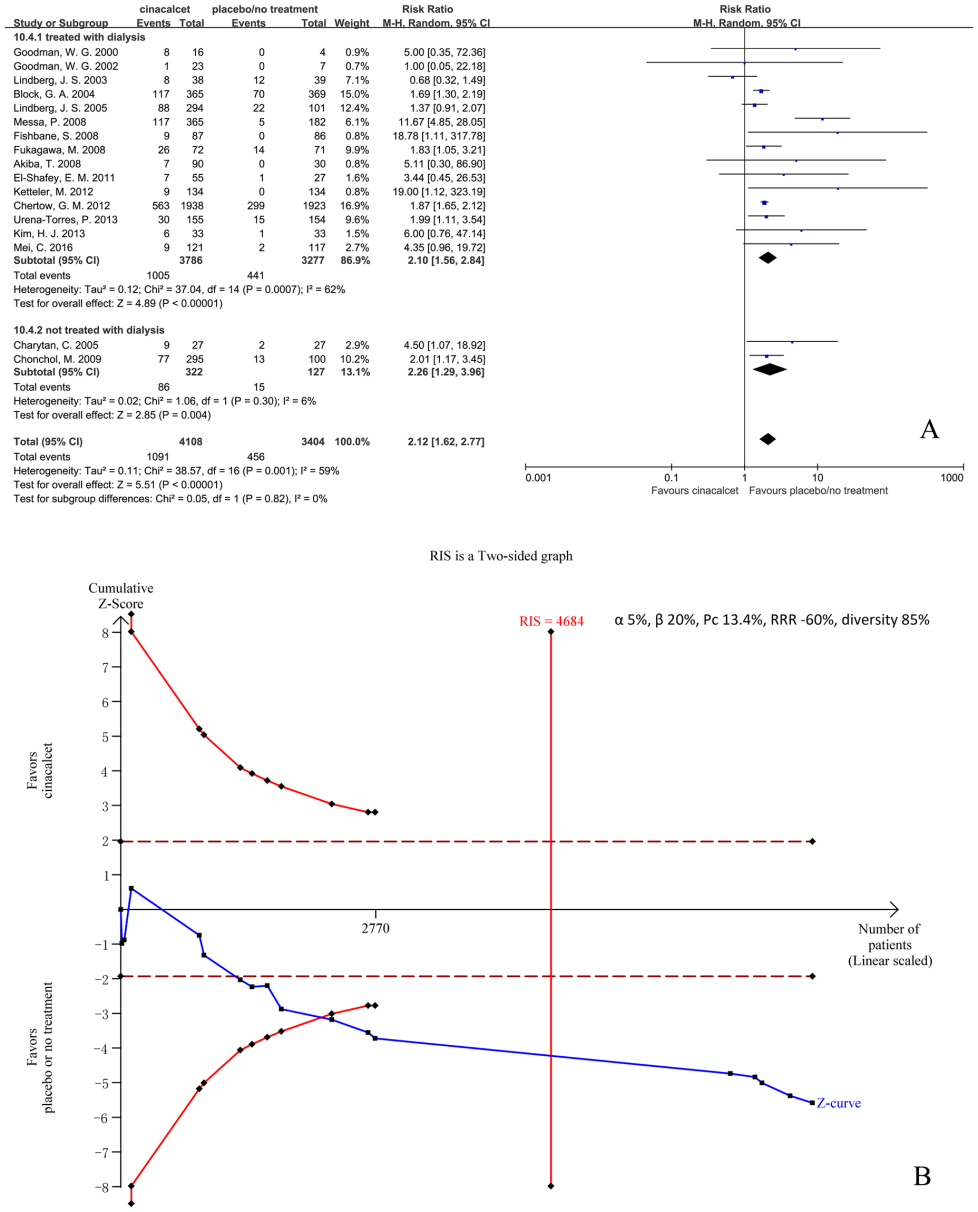
Forest plot (**A**) and trial sequential analysis (**B**) on nausea in cinacalcet group versus control group. RRR: relative risk reduction, Pc: event proportion in control group.

Seventeen trials with 7512 participants that had reported the incidence of nausea were included in our meta-analysis. The results showed that cinacalcet increased the risk of nausea (RR 2.12, 95% CI 1.62 to 2.77, P < 0.001), as determined using a random effects model (I^2^ = 59%, P = 0.001) (Fig. 9A). Our subgroup analysis showed that neither drug administration duration nor CKD staging had an influence on pooled RR (test for subgroup differences: I^2^ = 0%, P = 0.67 vs. I^2^ = 0%, P = 0.82). Our sensitivity analysis revealed that no single study had a significant influence on pooled RR (Figure S4, additional file 2).

RIS was 4684 participants. The cumulative Z curve (blue line) crossed both the conventional boundaries (black dotted line) and the trial sequential monitoring boundaries (red inward slash), and RIS has been reached (Fig. 9B). The TSA-adjusted 95% CI of RR was 1.45 to 3.04.

#### Vomiting

**Figure 10:**
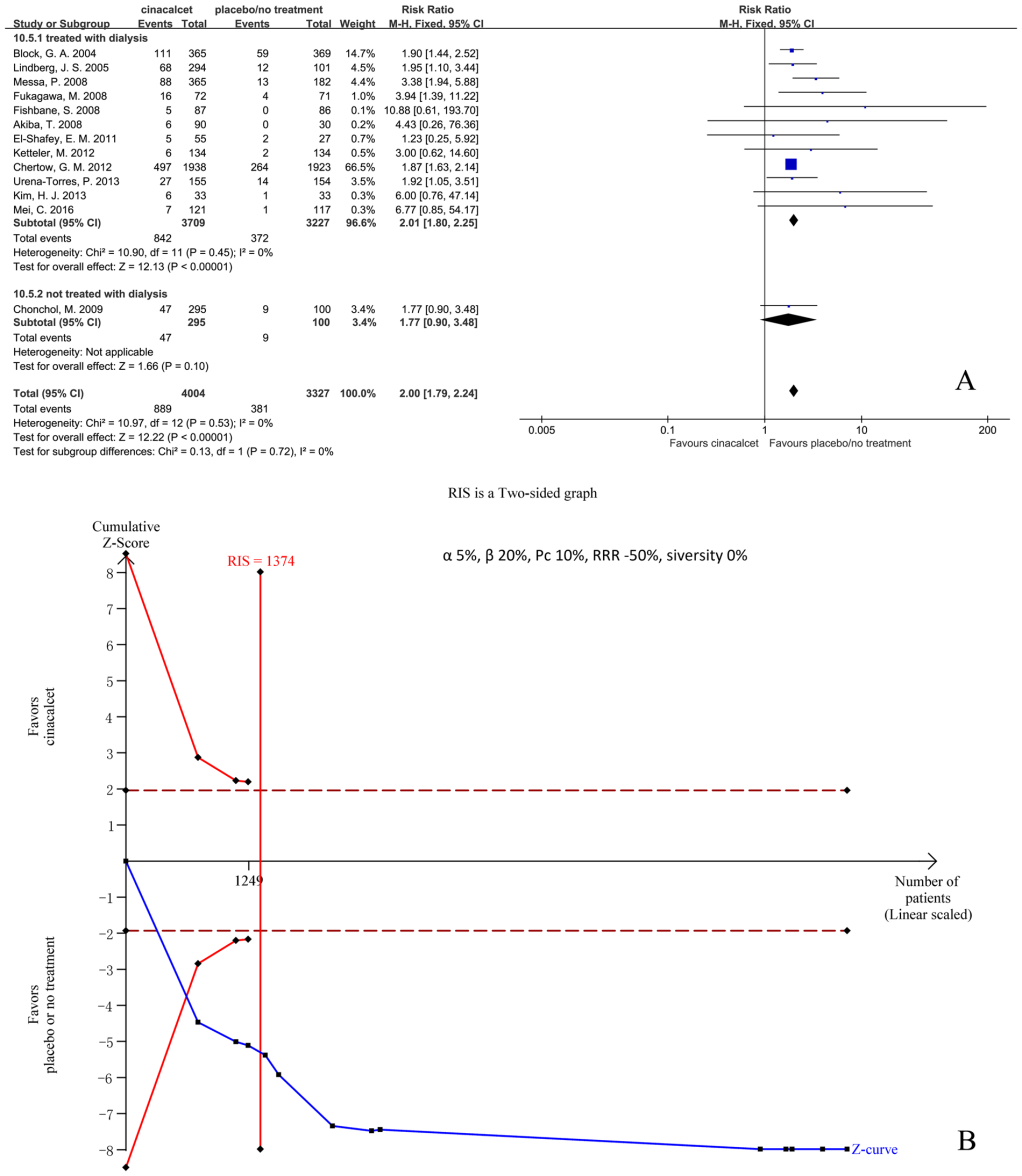
Forest plot (**A**) and trial sequential analysis (**B**) on vomiting in cinacalcet group versus control group. RRR: relative risk reduction, Pc: event proportion in control group.

Thirteen trials with 7331 participants that had reported the incidence of vomiting were included in our meta-analysis. The results showed that cinacalcet increased the risk of vomiting (RR 2.00, 95% CI 1.79 to 2.24, P < 0.001), as assessed using a fixed effects model (I^2^ = 0%, P = 0.53) (Fig. 10A).

RIS was 1374 participants. The cumulative Z curve (blue line) crossed both the conventional boundaries (black dotted line) and the trial sequential monitoring boundaries (red inward slash), and RIS has been reached (Fig. 10B). The TSA-adjusted 95% CI of RR was 1.77 to 2.26.

#### Diarrhea

**Figure 11:**
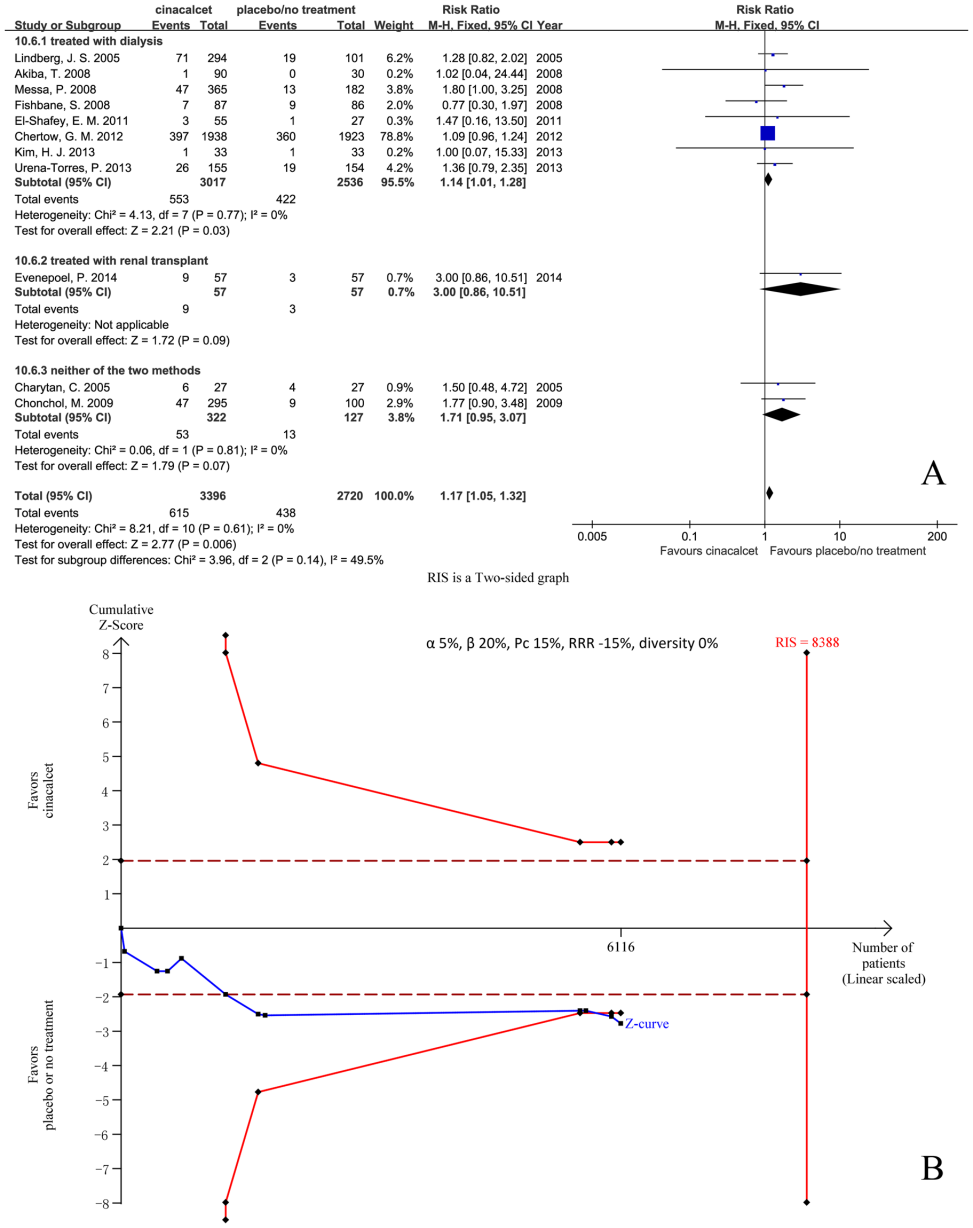
Forest plot (**A**) and trial sequential analysis (**B**) on diarrhoea in cinacalcet group versus control group. RRR: relative risk reduction, Pc: event proportion in control group.

Eleven trials with 6116 participants that had reported the incidence of diarrhea were included in our meta-analysis. The results showed that cinacalcet increased the risk of diarrhea (RR 1.17, 95% CI 1.05 to 1.32, P = 0.006), as determined using a fixed effects model (I^2^ = 0%, P = 0.61) (Fig. 11A).

RIS was 8388 participants. The cumulative Z curve (blue line) crossed the trial sequential monitoring boundaries (red inward slash) before the RIS has been reached (Fig. 11B). The TSA-adjusted 95% CI of RR was 1.02 to 1.36.

#### Muscle cramp or spasms

**Figure 12:**
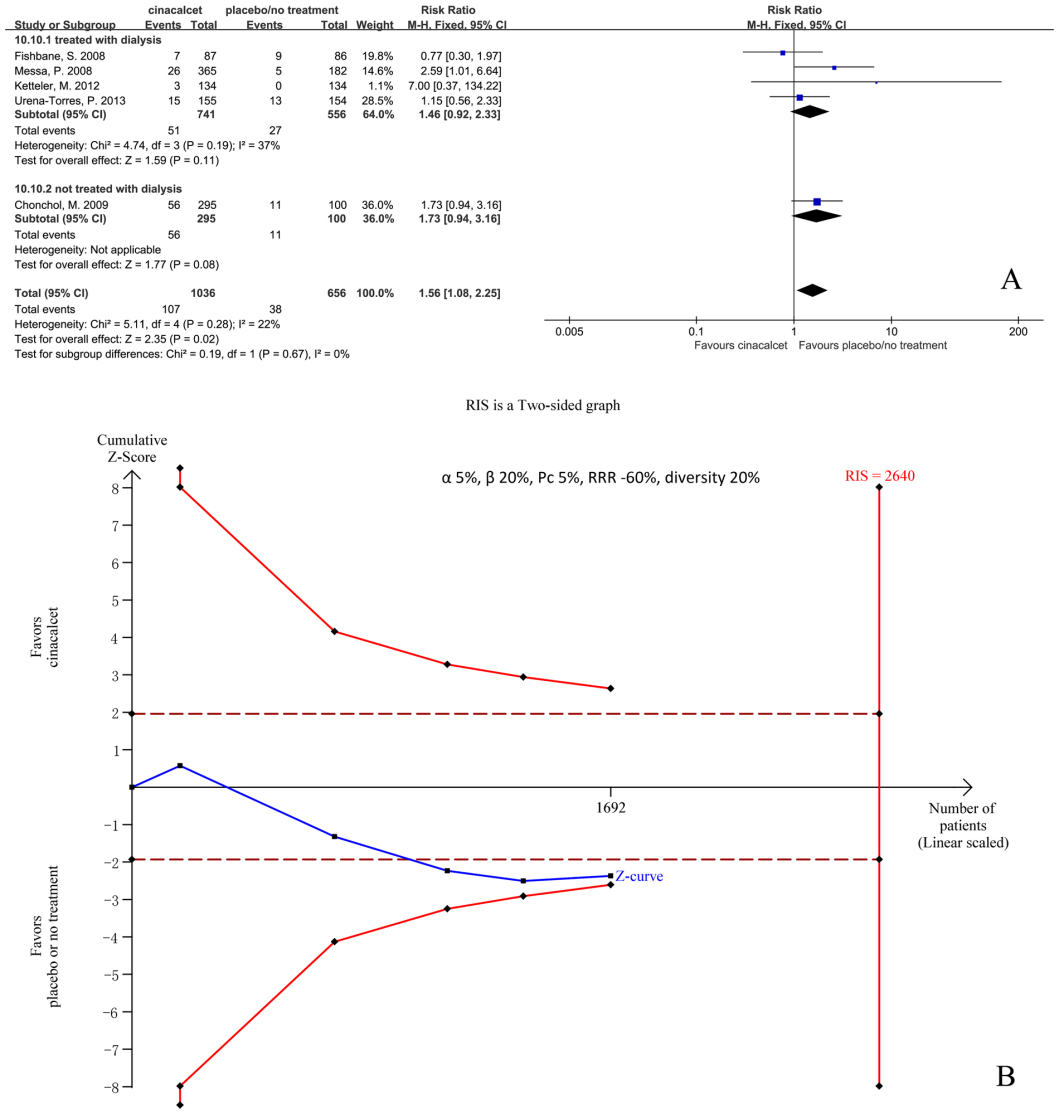
Forest plot (**A**) and trial sequential analysis (**B**) on muscle cramp or spasms in cinacalcet group versus control group. RRR: relative risk reduction, Pc: event proportion in control group.

Five trials with 1692 participants that had reported the incidence of muscle cramp or spasms were included in our meta-analysis. The results showed that cinacalcet increased the risk of muscle cramp or spasms (RR 1.56, 95% CI 1.08 to 2.25, P = 0.02), as determined using a fixed effects model (I^2^ = 22%, P = 0.28) (Fig. 12A).

RIS was 2640 participants, 64.1% of whom were accrued in our meta-analysis. The cumulative Z curve (blue line) crossed the conventional boundaries (black dotted line) but did not cross the trial sequential monitoring boundaries (red inward slash), and RIS has not been reached (Fig. 12B). The TSA-adjusted 95% CI of RR was 0.92 to 2.62.

#### Hypotension

**Figure 13:**
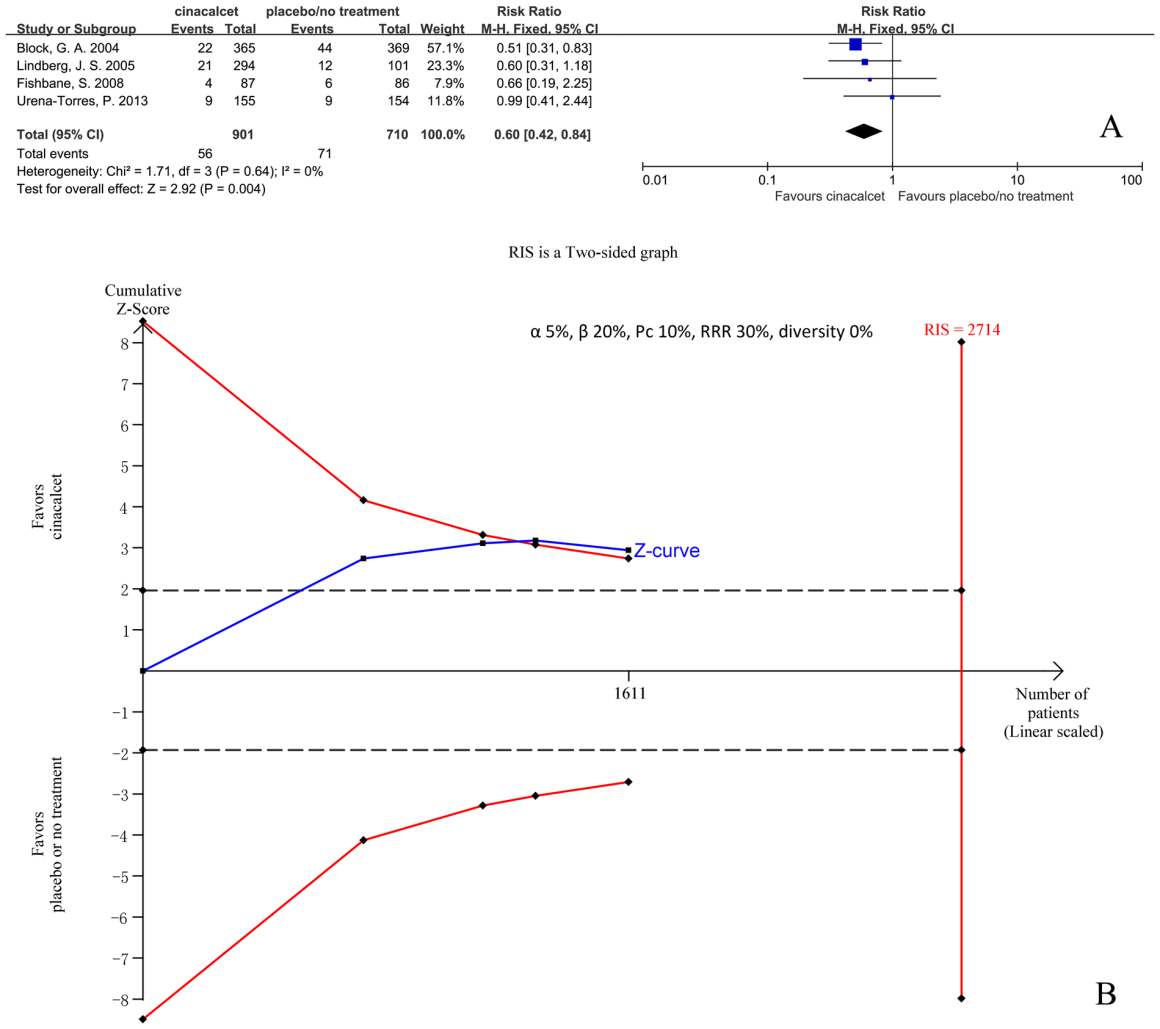
Forest plot (**A**) and trial sequential analysis (**B**) on hypotension in cinacalcet group versus control group. RRR: relative risk reduction, Pc: event proportion in control group.

Four trials with 1611 participants that had reported the incidence of hypotension were included in our meta-analysis. The results showed that cinacalcet decreased the risk of hypotension (RR 0.60, 95% CI 0.42 to 0.84, P = 0.004), as determined using a fixed effects model (I^2^ = 0%, P = 0.64) (Fig. 13A).

RIS was 2714 participants. The cumulative Z curve (blue line) crossed the trial sequential monitoring boundaries (red inward slash) before the RIS had been reached (Fig. 13B). The TSA-adjusted 95% CI of RR was 0.37 to 0.97. (All clinical outcomes are summarized in Table 1).

**Table 1:** Summary of effect of cinacalcet on main outcomes.

Outcomes	subgroup	No of patients (studies)	RR [95% CI]	p for RR	heterogeneity I^2%^ (p)	effects model	RIS	Description of TSA graph: cumulative Z curve crossed the TSA boundaries
all-cause mortality	all	8386 (21)	0.97 [0.89, 1.05]	0.41	0 (0.95)	fixed	5260	no
	dialysis	7814 (18)	0.97 [0.89, 1.05]	0.47	0 (0.97)	fixed	5260	no
	renal transplant	114 (1)	3.00 [0.12, 72.13]	0.50	NA	fixed	NA	NA
	none of two	458 (2)	0.28 [0.05, 1.44]	0.13	0 (0.77)	fixed	NA	NA
cardiovascular mortality	all	5418 (12)	0.95 [0.83, 1.07]	0.39	0 (0.46)	fixed	3780	no
	dialysis	4846 (9)	0.96 [0.84, 1.08]	0.48	0 (0.47)	fixed	3780	no
	renal transplant	114 (1)	NA	NA	NA	fixed	NA	NA
	none of two	458 (2)	0.28 [0.05, 1.44]	0.13	0 (0.77)	fixed	NA	NA
parathyroidectomy	dialysis	5488 (7)	0.48 [0.40, 0.59]	<0.00001	0 (0.69)	fixed	5787	yes
fractures	all	4053 (3)	0.58 [0.21, 1.59]	0.29	48 (0.14)	random	76376	no
	dialysis	3939 (2)	0.53 [0.12, 2.25]	0.39	72 (0.06)	random	NA	NA
	renal transplant	114 (1)	0.50 [0.05, 5.36]	0.57	NA	random	NA	NA
all adverse events	all	7685 (15)	1.04 [1.00, 1.09]	0.03	68 (<0.0001)	random	19152	no
	dialysis	7236 (13)	1.04 [1.00, 1.09]	0.07	71 (<0.0001)	random	22982	no
	none of two	449 (2)	1.07 [1.00, 1.14]	0.07	0 (0.38)	random	NA	NA
hypocalcaemia	all	7785 (18)	8.48 [6.37, 11.29]	<0.00001	0 (0.81)	fixed	6522	yes
	dialysis	7336 (16)	8.37 [6.26, 11.49]	<0.00001	0 (0.71)	fixed	6522	yes
	none of two	449 (2)	11.15 [2.12, 58.52]	0.004	0 (0.87)	fixed	NA	NA
hypercalcaemia	dialysis	4971 (5)	0.40 [0.11, 1.52]	0.18	79 (0.0008)	random	50437	no
nausea	all	7512 (17)	2.12 [1.62, 2.77]	<0.00001	59 (0.001)	random	4684	yes
	dialysis	7063 (15)	2.10 [1.56, 2.84]	<0.00001	62 (0.0007)	random	5599	no
	none of two	449 (2)	2.26 [1.29, 3.96]	0.004	6 (0.30)	random	NA	NA
vomiting	all	7331 (13)	2.00 [1.79, 2.24]	<0.00001	0 (0.53)	fixed	1374	yes
	dialysis	6936 (12)	2.01 [1.80, 2.245	<0.00001	0 (0.45)	fixed	1374	yes
	none of two	395 (1)	1.77 [0.90, 3.48]	0.10	NA	fixed	NA	NA
diarrhoea	all	6116 (11)	1.17 [1.05, 1.32]	0.006	0 (0.61)	fixed	8388	yes
	dialysis	5553 (8)	1.14 [1.01, 1.28]	0.03	0 (0.77)	fixed	8388	no
	renal transplant	114 (1)	3.00 [0.86, 10.51]	0.09	NA	fixed	NA	NA
	none of two	449 (2)	1.71 [0.95, 3.07]	0.07	0 (0.81)	fixed	NA	NA
muscle cramp or spasms	all	1692 (5)	1.56 [1.08, 2.25]	0.02	22 (0.68)	fixed	2640	no
	dialysis	1297 (4)	1.46 [0.92, 2.33]	0.11	37 (0.19)	fixed	3518	no
	none of two	395 (1)	1.73 [0.94, 3.16]	0.08	NA	fixed	NA	NA
hypotension	dialysis	1611 (4)	0.60 [0.42, 0.84]	0.004	0 (0.64)	fixed	2714	yes

NA: not available, RR: risk ratio, CI: confidence intervals, RIS: required information size, TSA: trial sequential analysis.

#### Other adverse events

The effect of cinacalcet on the adverse events of constipation, abdominal pain, dyspepsia, asthenia/fatigue or muscle weakness/paresthesia, upper respiratory tract infection, dyspnea, and headache was uncertain (data were not shown).

### Biochemical parameters

Our results showed that cinacalcet reduced the levels of serum PTH, calcium, phosphorus, and calcium by phosphorus product in patients under dialysis treatment of. However, our results indicated that serum phosphorus levels were much lower in the control group. More details are shown in Table 2.

**Table 2:** Summary of effect of cinacalcet on biochemical parameters.

Outcomes	subgroup	No of patients (studies)	RR or SMD [95% CI]	p for RR or SMD	heterogeneity I^2%^ (p)	effects model	heterogeneity for subgroup I^2%^ (p)	RIS	Description of TSA graph: cumulative Z curve crossed the TSA boundaries
PTH reduction ≥ 30%	all	3683 (14)	2.46 [1.59, 3.81]	<0.0001	95 (<0.00001)	random	0 (0.76)^a^	2092	yes
	dialysis	3237 (12)	2.42 [1.48, 3.97]	0.0004	96 (<0.00001)	random		2403	yes
	none of two	446 (2)	2.65 [1.96, 3.58]	<0.00001	0 (0.76)	random		NA	NA
achieved serum PTH target	all	3589 (13)	3.03 [1.78, 5.17]	<0.0001	94 (<0.00001)	random	83.5 (0.01)^a^	2293	yes
	dialysis	3197 (12)	2.74 [1.61, 4.67]	0.0002	93 (<0.00001)	random		2294	yes
	none of two	392 (1)	10.98 [4.17, 28.92]	<0.00001	NA	random		NA	NA
end of treatment serum PTH	all	3100 (12)	−0.67 [−0.90, −0.44]	<0.00001	88 (<0.00001)	random	0 (0.65)	1272	yes
	dialysis	2785 (10)	−0.68 [−0.95, −0.41]	<0.00001	90 (<0.00001)	random	0 (0.84)^a^	1370	yes
	renal transplant	114 (1)	−0.50 [−0.88, −0.13]	0.008	NA	random	0 (0.44)^b^	NA	NA
	none of two	201 (1)	−0.73 [−1.04, −0.41]	<0.00001	NA	random	0 (0.38)^c^	NA	NA
end of treatment serum calcium (Ca)	all	2623 (14)	−1.07 [−1.30, −0.84]	<0.00001	84 (<0.00001)	random	93.5 (<0.00001)	399	yes
	dialysis	2176 (11)	−0.93 [−1.13, −0.74]	<0.00001	73 (0.0001)	random	0 (0.5)^a^	314	yes
	renal transplant	114 (1)	−2.41 [−2.90, −1.92]	<0.00001	NA	random	96.7 (<0.00001)^b^	NA	NA
	none of two	333 (2)	−1.13 [−1.66, −0.60]	<0.0001	58 (0.12)	random	91.8 (0.0005)^c^	NA	NA
end of treatment serum phosphorous (P)	all	3366 (14)	−0.03 [−0.24, 0.17]	0.75	86 (<0.00001)	random	97.4 (<0.00001)	51194	no
	dialysis	2918 (11)	−0.21 [−0.29, −0.14]	<0.00001	26 (0.20)	fixed	96.9 (<0.00001)^a^	1937	yes
	renal transplant	114 (1)	1.28 [0.88, 1.69]	<0.00001	NA	random	98 (<0.00001)^b^	NA	NA
	none of two	334 (2)	0.53 [0.29, 0.77]	<0.0001	0 (0.83)	fixed	90 (0.002)^c^	NA	NA
end of treatment calcium by phosph- orous product (Ca*P)	all	3005 (11)	−0.49 [−0.67, −0.32]	<0.00001	77 (<0.00001)	random	97 (<0.00001)^a^	2606	yes
	dialysis	2613 (10)	−0.57 [−0.65, −0.49]	<0.00001	9 (0.36)	fixed		293	yes
	none of two	392 (1)	0.15 [−0.08, 0.38]	<0.00001	NA	random		NA	NA
Achieved (Ca*P) target	dialysis	1223 (5)	1.33 [1.20, 1.47]	<0.00001	45 (0.12)	fixed		1470	yes

NA: not available, a: subgroup dialysis vs subgroup none of two, b: subgroup dialysis vs renal transplant, c: subgroup renal transplant vs none of two, RR: risk ratio, SMD: standardized mean difference, CI: confidence intervals, RIS: required information size, TSA: trial sequential analysis.

### Bone turnover markers

Cinacalcet had an uncertain effect on the level of serum BALP (SMD −0.00, 95% CI −0.31 to 0.31, P = 0.98, I^2^ = 47%, 4 trials, n = 343), osteocalcin (SMD −0.34, 95% CI −0.96 to 0.28, P = 0.28, I^2^ = 86%, 3 trials, n = 311) and urine NTx (SMD −0.10, 95% CI −0.38 to 0.18, P = 0.48, I^2^ = 27%, 3 trials, n = 200) (Figures S5–7, additional file 2:).

#### Publication bias

Begg’s funnel plot showed no publication bias (Figures S8–15, additional file 2:).

## Discussion

Our updated meta-analysis revealed that cinacalcet did not reduce all-cause mortality and cardiovascular mortality in patients with SHPT caused by CKD, and TSA results showed that our outcomes were reliable and no more randomized controlled trials are required. Our meta-analysis showed that cinacalcet may reduce the proportion of patients that required parathyroidectomy compared with placebo and this may be a potential suggestion to clinicians when parathyroidectomy was not the best treatment for patients. We also found that the use of cinacalcet was correlated with a relatively high risk of adverse events, including hypocalcemia, nausea, vomiting and diarrhea. The absolute risk of hypocalcemia, nausea, vomiting and diarrhea increased by 10.31%, 13.16%, 10.57% and 2.01%, respectively compared with placebo or no treatment. Additionally, the absolute risk of hypotension decreased by 3.78%. Our TSA analysis showed that these results were reliable and no further randomized controlled trials are required. Although Cunningham *et al*. have reported that cinacalcet reduced the fracture rate^38^, our meta-analysis did not show a reduction in the incidence of fractures. However, our analysis was substantially underpowered (only 5.3% RIS was achieved) and thus further randomized controlled trials are required. Our findings were based on the content of moderate to high quality of the included trials by GRADE, and heterogeneity between trials was small.

Although cinacalcet is widely used in clinical practice, we suggest that it should not be used in conventional therapy because it does not improve mortality and may cause various drug-related adverse events. Of note, the duration time of drug administration was within one year in all included trials except one trial conducted by Chertow *et al*.^22^. Although Chertow *et al*. reported no statistically significant reduced risk of death during their five-year study and follow-up period, their lag-censoring analysis at 6 months after study-drug discontinuation showed a significant reduction in all-cause mortality in the cinacalcet group (hazard ratio, 0.83 [95% CI, 0.73 to 0.96]). Therefore, it is possible that cinacalcet may reduce long-term mortality, indicating that additional high quality trials with large sample sizes investigating effect of cinacalcet on long-term mortality are required. It is well established that some drug adverse effects are transient, thus it is likely that the adverse effects of cinacalcet will be eliminated over time.

We found an obvious beneficial effect of cinacalcet on serum PTH and calcium levels. The effect of cinacalcet on the level of serum phosphorus and calcium-by-phosphorus product seemed to be associated with the CKD stage. Cinacalcet apparently reduced the level of serum phosphorus and calcium-by-phosphorous product in patients with stage 5 CKD treated with dialysis. In contrast, our results showed that cinacalcet treatment was less effective compared to the control treatment for patients with stage 3–4 CKD without dialysis treatment. Due to the paucity of information regarding the effect of cinacalcet in patients with stage 3–4 CKD, our results are likely to be unreliable and more trials are required.

The majority of clinical trials and clinical practice have indicated biological markers (serum PTH, calcium, and phosphorus) as primary endpoints for the assessment of drug efficacy because they are easily measurable, respond to intervention before patient-level outcomes become evident (such as mortality), and the effect of intervention on these markers is associated with the effect on patient-level outcomes^39^. The NICE Clinical practice guidelines suggested that cinacalcet should be administered when the levels of serum PTH are very high. However, Palmer *et al*. reported that the relationship between the effect of cinacalcet on biochemical parameters and all-cause and cardiovascular mortality was weak and imprecise^13^, in accordance with our results. In addition, cinacalcet increased the occurrence of adverse events (such as hypocalcemia and gastrointestinal disorders). Thus, we raise concerns regarding the suitability of biological markers as primary endpoints and the clinical efficiency of cinacalcet on patients with very high serum PTH levels. We suggest that biological markers do not seem to be sufficiently valid parameters for the assessment of drug efficacy and that in clinical practice the condition of patients should not be assessed based on the results of biological markers only.

Bone remodeling is dependent on the dynamic balance between bone formation and bone resorption. Excessively high level of serum PTH leads to imbalance of these two processes and affects bone and mineral metabolism. BALP, osteocalcin and urine NTx serve as biochemical markers for bone formation and bone resorption^40^. Some studies showed that cinacalcet may reduce serum BALP and osteocalcin levels. However, our results are not conclusive, because the trials included in our meta-analysis were of small size.

Our meta-analysis has several limitations. First, one must be cautious regarding the interpretation of parathyroidectomy because RIS was not reached according to our TSA results and surgical removal of the parathyroid glands is dependent on the treating clinician and is a human decision. Second, the duration time of drug treatment was within one year except one trial of five years, therefore it was impossible to assess the effect of cinacalcet on long-term mortality. Third, most included studies provided the range of doses, thus the mean dose of cinacalcet cannot be obtained. It is possible that the different doses of cinacalcet used in different studies may influence our results. Fourth, parallel randomized clinical trials that had compared cinacalcet with placebo or no treatment (did not use cinacalcet and placebo) were included. Vitamin D and phosphate binders were used in both groups in most studies. However, dose and adoption proportion of vitamin D and phosphate binders were various in some studies, thus likely influencing our results. Finally, there was substantial heterogeneity among the studies regarding the levels of the biochemical parameters and adverse events, which did not improve after sensitivity and subgroup analyses performed to examine the derivation of heterogeneity.

Substantial heterogeneity and insufficient sample size may account for the decreased level of evidence. Several potential factors were likely associated with the high degree of heterogeneity. First, because the data we extracted were aggregated but individual, certain baseline characteristics, such as age, sex, serum PTH level, duration and method of dialysis, medical history and medication history were not taken into consideration. Furthermore, the methods of measurement of the biochemical parameters may vary between laboratories. Finally, some adverse events may be subjective and are affected by other factors, such as the emotional condition of the patients.

In conclusion, our meta-analysis results showed that cinacalcet not only did not reduce all-cause mortality and cardiovascular mortality but increased the risk of adverse events, such as hypocalcemia and gastrointestinal disorders (nausea, vomiting and diarrhea), although it improved serum PTH, calcium and phosphorus levels. Further randomized controlled trials investigating the effect of cinacalcet on long-term mortality are required.

## Materials and Methods

### Protocol and Registration

Our meta-analysis of randomized controlled trials was performed according to the Preferred Reporting Items for Systematic reviews and meta-analyses (PRISMA) recommendations (additional file 1)^41^. A protocol for this meta-analysis has been registered on PROSPERO (http://www.crd.york.ac.uk/prospero) and the registration number is: CRD42016036585.

### Search strategy

For this meta-analysis update, we conducted a search of Pubmed, Embase, and the Cochrane Central Register of Controlled Trials (CENTRAL) (all up to March 2016), according to the guidelines of the Cochrane Handbook^42^, without language restrictions. We used the following subject headings and keywords: “kidney diseases”, “chronic kidney failure”, “kidney failure”, “renal failure”, “renal dialysis”, “kidney dialysis”, “dialysis”, “hemodialysis”, “haemodialysis”, “peritoneal dialysis”, “CAPD”, “CCPD”, “APD”, “secondary hyperparathyroidism”, “cinacalcet”, “mimpara”, “sensipar”, “calcimimetic”, “calcimimetic agent”, “R-568”, “R-467”, “AMG 074”, “AMG 073”, “KRN 1493”, “naphthalene derivative”, “naphthalene” *et al*. A supplementary search of the reference lists from all retrieved trials and reviews was also performed. In case the articles were not available from databases, we directly contacted the corresponding authors by mail. All results were imported into Endnote X7 (Thomson Reuters, New York, USA) for the exclusion of duplicates, and subsequently, we screened the titles, abstracts and full-texts of eligible trials.

### Inclusion and exclusion criteria

We included parallel randomized clinical trials that had compared cinacalcet with placebo or no treatment (did not use cinacalcet and placebo) in patients ≥18 years old with SHPT caused by CKD. In a single study, the diagnosis of CKD was in accordance with the National Kidney Foundation Kidney Disease Outcomes Quality Initiative (NKF-K/DOQI). NKF-K/DOQI defined CKD as an abnormality of structure and function of kidney for 3 months or more with estimated glomerular filtration rate (GFR) below 60 mL/min per 1.73 m^2^ irrespective of kidney damage^43^. In addition, serum intact PTH (iPTH) levels should meet the following criteria: greater than 250–300 pg/mL in stage 5 CKD (GFR < 15 mL/min per 1.73 m^2^) treated with dialysis or greater than 100–160 pg/mL in stage 3–4 CKD (GFR 15–60 mL/min per 1.73 m^2^).

### Endpoints and data extraction

The primary endpoints were all-cause mortality, cardiovascular mortality, parathyroidectomy, fractures, total adverse events and drug-related adverse events (hypocalcemia, hypercalcemia, nausea, vomiting, diarrhea, constipation, abdominal pain, dyspepsia, muscle cramp/spasms, asthenia/fatigue or muscle weakness/paresthesia, upper respiratory tract infection, dyspnea, headache and hypotension). The secondary endpoints included fulfillment of the PTH level target (iPTH ≤ 250/300 or 150 ≤ iPTH ≤ 300 pg/mL in stage 5 CKD, iPTH ≤ 110 pg/mL in stage 4 CKD, and iPTH ≤ 70 pg/mL in stage 3 CKD), a ≥ 30% reduction in PTH levels, end-of-treatment PTH level, end-of-treatment serum calcium level, end-of-treatment serum phosphorus level, fulfilment of calcium-by-phosphorus product target (Ca × P ≤ 5.5 mg^2^/dL^2^), end-of-treatment serum calcium-by-phosphorus product. Tertiary endpoints were end-of-treatment bone turnover markers (BALP, osteocalcin and urine NTx). Data including date of publication, name of first author, study type, dosing strategy of cinacalcet, the study period (dose-titration phase, maintenance phase and efficacy assessment phase), patient characteristics (mean age, age range, number of patients and sex ratio, country of origin), and the above endpoints were extracted from the eligible studies using a standard data extraction form.

Database search, eligibility evaluation and data extraction were performed independently by two authors (Guoqi Wang and Hongyan Liu); lack of consensus was resolved by a third author.

### Data synthesis and statistical analysis

RevMan software (version 5.1; Cochrane Collaboration, Copenhagen, Denmark) and STATA 12.0 (StatCorp, College Station,TX, USA) were used for Statistical analysis. Risk ratio (RR) with 95% confidence intervals (CIs) and standardized mean difference (SMD) with 95% CIs were used for dichotomous and continuous variables, respectively. Standard deviation (SD) was calculated according to the formula SD = SE × $\sqrt{n}$, when the data were expressed as mean ± SE (standard error of mean). A p value < 0.05 was considered as statistically significant. We assessed the heterogeneity between trials with the Cochran’s Q-statistic test and the I^2^ test, measuring the extent of inconsistency—derived from heterogeneity rather than chance—of the results from eligible studies^44^. The random-effects model from DerSimonian and Laird^45^ was adopted on the condition that I^2^ was more than 50% or P value (Q-test) was less than 0.05, or else a fixed-effects model was used. Subgroup analysis was performed according to CKD staging (stage 5 CKD patients treated with dialysis, stage 3–4 CKD patients not treated with dialysis, stage 1–3 CKD patients treated with renal transplant) and drug administration duration to establish the derivation of heterogeneity. Forest plots were completed in RevMan version 5.1 software. STATA 12.0 was used to perform sensitivity analysis by omitting any one study successively and to generate funnel plots to assess the constancy of total estimate and the publication bias, respectively.

### TSA

Similar to clinical trials, systematic reviews and meta-analysis studies require estimation of the sample size to reduce the risk of random errors and ensure the reliability of results^46^. TSA can control for the risks of type I and type II errors and calculate RIS, required by systematic review and meta-analysis^47^. When the cumulative Z curve crosses the trial sequential monitoring boundaries with or without the achievement of RIS, we think that the anticipated intervention effect may have been reached and no further trials are required. If RIS has been reached, but the cumulative Z curve crosses neither the trial sequential monitoring boundaries nor conventional boundaries, we think that there is no statistical difference between the two groups in comparison and no more trials are required. If the cumulative Z curve crosses the futility boundaries, we can also think that there is no difference between the two groups in comparison. However, if the cumulative Z curve does not cross the trial sequential monitoring boundaries and, at the same time, RIS has not been reached, we conclude that more trials are required.

We adopted a method of constant continuity correction for handling zero-event trials^48^, and added a continuity correction factor of 0.5 to the number of events and non-events in each group.

Two-sided tests, a type I error of 5% and a type II error of 20% (a power of 80%) were used for calculating the RIS. For dichotomous data, the incidence in the control group was derived from the results of our meta-analysis, and a relative risk reduction or increase was estimated according to the information from related areas.

### Risk of bias and quality of evidence

Two authors independently assessed the following seven categories of risk of bias according to the Cochrane guidelines^42^ and lack of consensus was resolved in group discussions. The risk of bias was classified in the following seven categories: (1) random sequence generation, (2) allocation concealment, (3) blinding of participants and personnel, (4) blinding of outcome assessment, (5) incomplete outcome data, (6) selective outcome reporting, (7) other sources of bias. Each category can be graded into three levels: low risk, unclear risk, or high risk. In addition, we evaluated and graded the quality of evidence for all endpoints of our meta-analysis in line with the Grading of Recommendations Assessment, Development and Evaluation (GRADE) guidelines^49^. The GRADE includes five aspects, which are risk of bias, inconsistency, indirectness, imprecision, and publication bias, and grades the evidence into four levels: very low, low, moderate, or high quality.

### Financial Disclosure

There are no sources of funding involved in this paper.

### Ethics approval and consent to participate

Ethics approval is not applicable. This study is a research on research study.

### Availability of data and materials

All data generated or analysed during this study are included in this article and its supplementary information files.

## Electronic supplementary material

Additional file 1

Additional file 2
