# Supplementary material for: Cinacalcet versus Placebo for secondary hyperparathyroidism in chronic kidney disease patients: a meta-analysis of randomized controlled trials and trial sequential analysis
Source: Sci Rep. 2018 Feb 15;8:3111. doi: 10.1038/s41598-018-21397-8 (PMC5814442; doi:10.1038/s41598-018-21397-8)
Supplement: Supplementary file 2 — Additional file 2 [file 41598_2018_21397_MOESM2_ESM.pdf]

Additional File 2

Cinacalcet versus Placebo for secondary hyperparathyroidism in chronic kidney disease patients: a meta-analysis of randomized controlled trials and trial sequential analysis

Guoqi Wang[1$], Hongyan Liu[2$], Chengzhi Wang[2], Xiaojian Ji[3], Weijun Gu[2*], Yiming Mu[2*]

1. Department of Orthopedics, Chinese PLA General Hospital, No. 28 Fuxing Road, Haidian District, Beijing 100853, P.R. China,

2. Department of Endocrinology, Chinese PLA General Hospital, No. 28 Fuxing Road, Haidian District, Beijing 100853, P.R. China,

3. Department of rheumatology, Chinese PLA General Hospital, No. 28 Fuxing Road, Haidian District, Beijing 100853, P.R. China

* Correspondence:

Yiming Mu, E-mail: muyiming@301hospital.com.cn Tel.: +86 10 55499001; Fax: +86 10 68168917;

Weijun Gu, E-mail: guweijun301@163.com Tel.: +86 10 55499001; Fax: +86 10 68168917;

[$] These authors contributed equally to this work.

Table S1 characteristics of studies included

study	intervention		Co-interventions		dialysis	duration (week)	number	mean age (year) mean (SD)		sex (male/female)	
	cinacalcet	control	cinacalcet	control			cinacalcet *V* control	cinacalcet	control	cinacalcet	control
Mei, C. (2016)	25-100 mg/d	placebo	Vitamin D (68.6%)<br>Phosphate binders (62.7%)	Vitamin D (67.5%)<br>Phosphate binders (63.2%)	HD	14	121 *V* 117	50.02 (11.17)	50.12 (11.34)	68/50	66/48
Wetmore, J. B. (2015)	30-180 mg/d	vitamin D#	Phosphate binders (90.3%)	Phosphate binders (91.7%)	HD	52	153 *V* 155	53 (21–81)[a]	55 (22–86)[a]	93/62	95/62
Evenepoel, P. (2014)	30-180 mg/d	placebo	NA	NA	kidney transplant	52	57 *V* 57	53.0 (10.7)	51.7 (9.9)	31/26	32/25
Kim, H. J. (2013)	25-50 mg/d	vitamin D#	Vitamin D<br>Phosphate binders (90.9%)	Vitamin D<br>Phosphate binders (81.8%)	PD	16	33 *V* 33	48.8 (11.5)	47.2 (8.4)	20/13	15/18
Urena-Torres, P. (2013)	30-180 mg/d<br>Low dose vitamin D	vitamin D#	Phosphate binders	Phosphate binders	HD	52	155 *V* 154	57.9 (13.6)	57.0 (14.6)	83/70	95/56
Ketteler, M. (2012)	Low dose vitamin D	vitamin D#	NA	NA	HD	28	134 *V* 134	59.9 (12.0) (iv)<br>65.1 (12.5) (oral)	61.2 (12.7) (iv)<br>65.7 (13.5) (oral)	81/53	87/47
Chertow, G. M. (2012)	30-180 mg/d	placebo	Vitamin D (59.3%);<br>Phosphate binders (87.8%	Vitamin D (59.6%);<br>Phosphate binders (89%)	HD	260	1948 *V* 1935	55 (35.0, 74.0)[b]	54 (35.0, 73.0)[b]	1140/808	1167/768
El-Shafey, E. M. (2011)	30-180 mg/d	conventional therapy	Vitamin D (50%);<br>Phosphate binders (86%)	Vitamin D (52%);<br>Phosphate binders (89%)	HD	36	55 *V* 27	51.5 (12.7)	51.8 (15)	27/28	14/13
Raggi, P. (2011)	30-180 mg/d<br>Low dose vitamin D	vitamin D#	Vitamin D (75%);<br>Phosphate binders	Vitamin D (79%);<br>Phosphate binders	HD	52	180 *V* 180	61.2 (12.6)	61.8 (12.8)	112/68	95/85
Chonchol, M.	30-180 mg/d	placebo	Vitamin D (21%);	Vitamin D (21%);	none	22	302 *V* 102	64.7 (13.3)	66.2 (12.2)	177/125	60/42

(2009)			Phosphate binders (19%)	Phosphate binders (18%)							
Messa, P. (2008)	30-180 mg/d	placebo	Vitamin D (68%); Phosphate binders (92%)	Vitamin D (68%); Phosphate binders (90%)	HD	23	368 *V* 184	58.5 (14.5)	58.3 (14.5)	224/144	117/67
Malluche, H. H. (2008)	30-180 mg/d	placebo	Vitamin D (47%); Phosphate binders (100%)	Vitamin D (54%); Phosphate binders (77%)	HD	52	32 *V* 16	50.3 (13.3)	51.5 (14.1)	12/7	9/4
Fukagawa, M. (2008)	25-100 mg/d	placebo	Vitamin D (87.5%); Phosphate binders (93.1%)	Vitamin D (88.7%); Phosphate binders (95.8%)	HD	14	72 *V* 71	54.7 (11	55.7 (11.7)	40/32	37/34
Akiba, T. (2008)	12.5,25,50mg/d	placebo	Vitamin D (67%); Phosphate binders (96%)	Vitamin D (70%); Phosphate binders (100%)	HD	3	91 *V* 30	56.7(9.2),55.8(7.7), 53.2(7.0)	51.8 (7.5)	54/25	25/5
Fishbane, S. (2008)	30-180 mg/d Low dose vitamin D	vitamin D[#]	NA	NA	HD	27	87 *V* 86	57.7 (14.9)	59 (12.4)	52/35	45/41
Lindberg, J. S. (2005)	30-180 mg/d	placebo	Vitamin D (65%); Phosphate binders (NA)	Vitamin D (69%); Phosphate binders (NA)	HD and PD	26	294 *V* 101	51.8 (14.0)	53.5 (13.9)	181/113	64/37
Charytan, C. (2005)	30-180 mg/d	placebo	Vitamin D (22%); Phosphate binders (37%)	Vitamin D (33%); Phosphate binders (48%)	none	18	27 *V* 27	60.6 (15.6)	61.9 (15.1)	16/11	22/5
Harris, R. Z. (2004)	25-300 mg/d	placebo	Vitamin D (71%); Phosphate binders (100%)	Vitamin D (60%); Phosphate binders (80%)	HD	12	17 *V* 5	48.5 (10.4)	48 (13.1)	14/3	4/1
Block, G. A. (2004)	30-180 mg/d	placebo	Vitamin D (66%); Phosphate binders (92%)	Vitamin D (67%); Phosphate binders (93%)	HD	26	371 *V* 370	54 (14)	55 (15)	226/145	229/141
Quarles, L. D. (2003)	25-100 mg/d	placebo	Vitamin D (61%); Phosphate binders (100%)	Vitamin D (69%); Phosphate binders (94%)	HD	18	36 *V* 35	49.6 (8.5)	47.9 (14.2)	27/9	17/18
Lindberg, J. S. (2003)	10-50 mg/d	placebo	Vitamin D (67%); Phosphate binders (87%)	Vitamin D (62%); Phosphate binders (87%)	HD	18	38 *V* 39	52.7 (16.4)	48.8 (15.6)	24/15	22/17
Goodman, W. G. (2002)	10,25,50 mg/d	placebo	NA	NA	HD	1	23 *V* 7	46 (16)		NA	NA

Goodman, W. G. (2000)	100 mg/d	placebo	Vitamin D (38%); Phosphate binders (31%)	Vitamin D (50%); Phosphate binders (50%)	HD	2	16 *V* 4	48.6 (12.4)	54.7 (16.8)	13/3	1/3

HD: hemodialysis, PD: peritoneal dialysis, NA: not applicable a: median (min to max), b: median (10th to 90th percentile), duration: the duration of drug treatment. None: none of hemodialysis or peritoneal dialysis, # the use of vitamin in two group is not comparable.

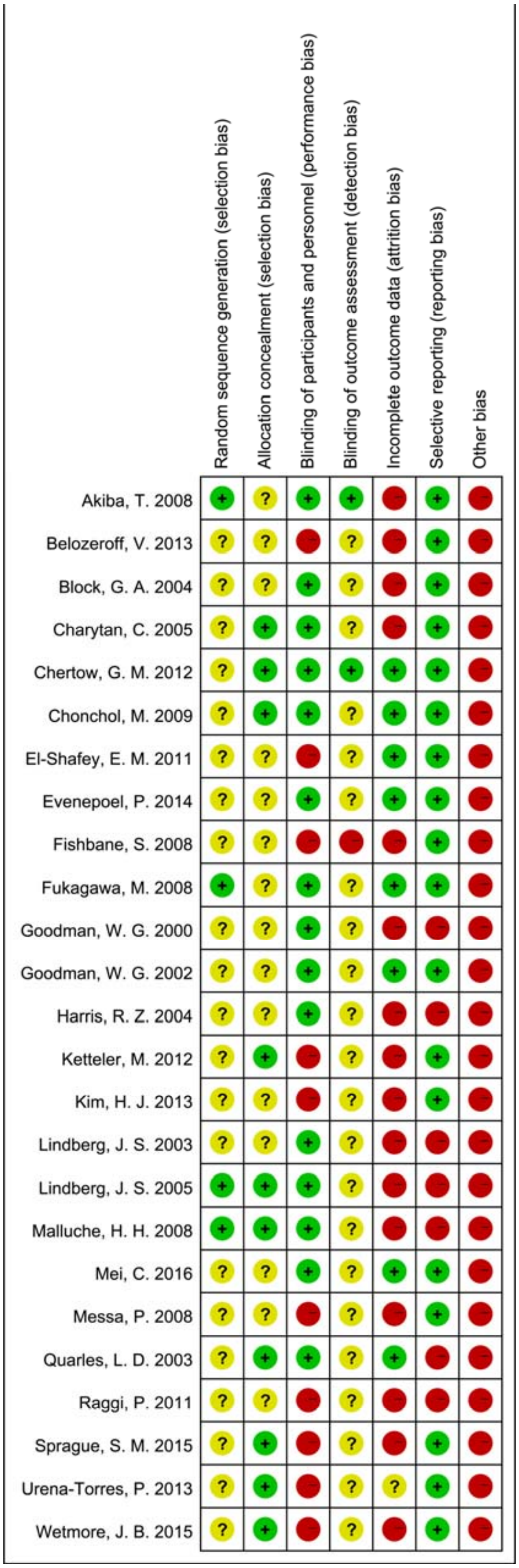

Figure S1 Risk of bias. green: low risk, yellow: unclear risk, red: high risk

Table S2 The GRADE evidence quality for outcomes

Outcomes	subgroup	No of patients (studies)	No with event/No in group (%)		RR or SMD [95% CI]	P	p for heterogeneity	I^2(%)	Quality
			cinacalcet	control					
all-cause mortality	all	8386 (21)	768/4557	776/3829	0.97 [0.89, 1.05]	0.41	0.95	0	high
	dialysis	7814 (18)	765/ 4171	772/ 3643	0.97 [0.89, 1.05]	0.47	0.97	0	high
	renal transplant	114 (1)	1/57	0/57	3.00 [0.12, 72.13]	0.50	NA	NA	
	none of two	458 (2)	2/329	4/129	0.28 [0.05, 1.44]	0.13	0.77	0	
cardiovascular mortality	all	5418 (12)	380/2862	397/2556	0.95 [0.83, 1.07]	0.39	0.46	0	high
	dialysis	4846 (9)	378/2476	393/2370	0.96 [0.84, 1.08]	0.48	0.47	0	high
	renal transplant	114 (1)	0/57	0/57	NA	NA	NA	NA	
	none of two	458 (2)	2/329	4/129	0.28 [0.05, 1.44]	0.13	0.77	0	
parathyroidectomy	dialysis	5488 (7)	142/2861	289/2627	0.48 [0.40, 0.59]	<0.00001	0.69	0	high
fracture	all	4053 (3)	241/2047	262/2006	0.58 [0.21, 1.59]	0.29	0.14	48	moderate[a]
	dialysis	3939 (2)	240/1990	260/1949	0.53 [0.12, 2.25]	0.39	0.06	72	Low[c]
	renal transplant	114 (1)	1/57	2/57	0.50 [0.05, 5.36]	0.57	NA	NA	
total adverse events	all	7685 (15)	3722/4173	3037/3512	1.04 [1.00, 1.09]	0.03	<0.0001	68	Low[c]
	dialysis	7236 (13)	3427/3851	2928/3385	1.04 [1.00, 1.09]	0.07	<0.0001	71	Low[c]
	none of two	449 (2)	295/322	109/127	1.07 [1.00, 1.14]	0.07	0.38	0	
hypocalcaemia	all	7785 (18)	479/4147	45/3638	8.48 [6.37, 11.29]	<0.00001	0.81	0	high
	dialysis	7336 (16)	440/3825	44/3511	8.37 [6.26, 11.19]	<0.00001	0.71	0	high
	none of two	449 (2)	39/322	1/127	11.15 [2.12, 58.52]	0.004	0.87	0	
hypercalcaemia	dialysis	4971 (5)	44/2494	75/2477	0.40 [0.11, 1.52]	0.18	0.0008	79	Low[c]
nausea	all	7512 (17)	1091/4108	456/3404	2.12 [1.62, 2.77]	<0.00001	0.001	59	moderate[b]

	dialysis	7063 (15)	1005/3786	441/3277	2.10 [1.56, 2.84]	<0.00001	0.0007	62	moderate[b]
	none of two	449 (2)	86/322	15/127	2.26 [1.29, 3.96]	0.004	0.30	6	
vomiting	all	7331 (13)	889/4004	381/3327	2.00 [1.79, 2.24]	<0.00001	0.53	0	high
	dialysis	6936 (12)	842/3709	372/3227	2.01 [1.80, 2.25]	<0.00001	0.45	0	high
	none of two	395 (1)	47/295	9/100	1.77 [0.90, 3.48]	0.10	NA	NA	
diarrhoea	all	6116 (11)	615/3396	438/2720	1.17 [1.05, 1.32]	0.006	0.61	0	high
	dialysis	5553 (8)	553/3017	422/2536	1.14 [1.01, 1.28]	0.03	0.77	0	high
	renal transplant	114 (1)	9/57	3/57	3.00 [0.86, 10.51]	0.09	NA	NA	
	none of two	449 (2)	53/322	13/127	1.71 [0.95, 3.07]	0.07	0.81	0	
muscle cramp or	all	1692 (5)	107/1036	38/656	1.56 [1.08, 2.25]	0.02	0.28	22	moderate[a]
spasms	dialysis	1297 (4)	51/741	27/556	1.46 [0.92, 2.33]	0.11	0.19	37	moderate[a]
	none of two	395 (1)	56/295	11/100	1.73 [0.94, 3.16]	0.08	NA	NA	
hypotension	dialysis	1611 (4)	56/901	71/710	0.60 [0.42, 0.84]	0.004	0.64	0	high
PTH reduction⩾30%	all	3683 (14)	1393/2130	416/1553	2.46 [1.59, 3.81]	<0.0001	<0.00001	95	moderate[b]
	dialysis	3237 (12)	1162/1810	383/1427	2.42 [1.48, 3.97]	0.0004	<0.00001	96	moderate[b]
	none of two	446 (2)	231/320	33/126	2.65 [1.96, 3.58]	<0.00001	0.76	0	
achieved serum PTH	all	3589 (13)	987/2076	263/1513	3.03 [1.78, 5.17]	<0.0001	<0.00001	94	moderate[b]
target	dialysis	3197 (12)	857/1783	259/1414	2.74 [1.61, 4.67]	0.0002	<0.00001	93	moderate[b]
	none of two	392 (1)	130/293	4/99	10.98 [4.17, 28.92]	<0.00001	NA	NA	
end of treatment serum	all	3100 (12)	1802	1298	-0.67 [-0.90, -0.44]	<0.00001	<0.00001	88	moderate[b]
PTH	dialysis	2785 (10)	1599	1186	-0.68 [-0.95, -0.41]	<0.00001	<0.00001	90	moderate[b]
	renal transplant	114 (1)	57	57	-0.50 [-0.88, -0.13]	0.008	NA	NA	
	none of two	201 (1)	146	55	-0.73 [-1.04, -0.41]	<0.00001	NA	NA	
end of treatment serum	all	2623 (14)	1584	1039	-1.07 [-1.30, -0.84]	<0.00001	<0.00001	84	moderate[b]
calcium	dialysis	2176 (11)	1295	881	-0.93 [-1.13, -0.74]	<0.00001	0.0001	73	moderate[b]
	renal transplant	114 (1)	57	57	-2.41 [-2.90, -1.92]	<0.00001	NA	NA	

	none of two	333 (2)	232	101	-1.13 [-1.66, -0.60]	<0.0001	0.12	58	
end of treatment serum	all	3366 (14)	1957	1409	-0.03 [-0.24, 0.17]	0.75	<0.00001	86	Low[c]
phosphorous	dialysis	2918 (11)	1667	1251	-0.22 [-0.31, -0.13]	<0.00001	0.20	26	high
	renal transplant	114 (1)	57	57	1.28 [0.88, 1.69]	<0.00001	NA	NA	
	none of two	334 (2)	233	101	0.53 [0.29, 0.77]	<0.0001	0.83	0	
end of treatment	all	3005 (11)	1806	1199	-0.49 [-0.67, -0.32]	<0.00001	<0.00001	77	moderate[b]
calcium by	dialysis	2613 (10)	1513	1100	-0.57 [-0.66, -0.48]	<0.00001	0.36	9	high
phosphorous product	none of two	392 (1)	293	99	0.15 [-0.08, 0.38]	<0.00001	<0.00001	77	
achieved (Ca*P) target	dialysis	1223 (5)	588/816	224/407	1.33 [1.20, 1.47]	<0.00001	0.12	45	high

a: total number<RIS, b I^2>50%, c total number<RIS, and I^2>50%, NA: not applicable

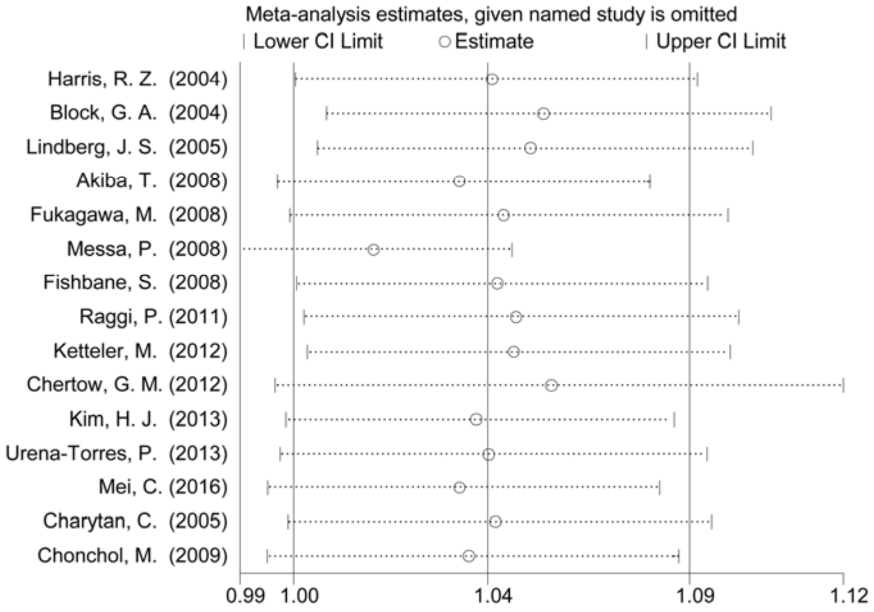

Figure S2. Sensitivity analysis of all advert events in cinacalcet group versus control group. CI= confidence interval

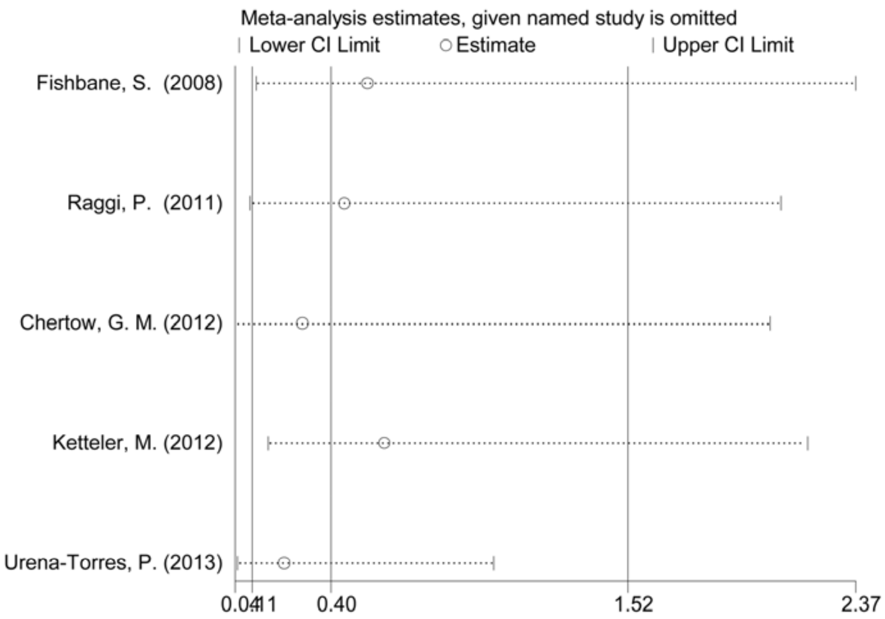

Figure S3. Sensitivity analysis of hypercalcaemia in cinacalcet group versus control group. CI= confidence interval

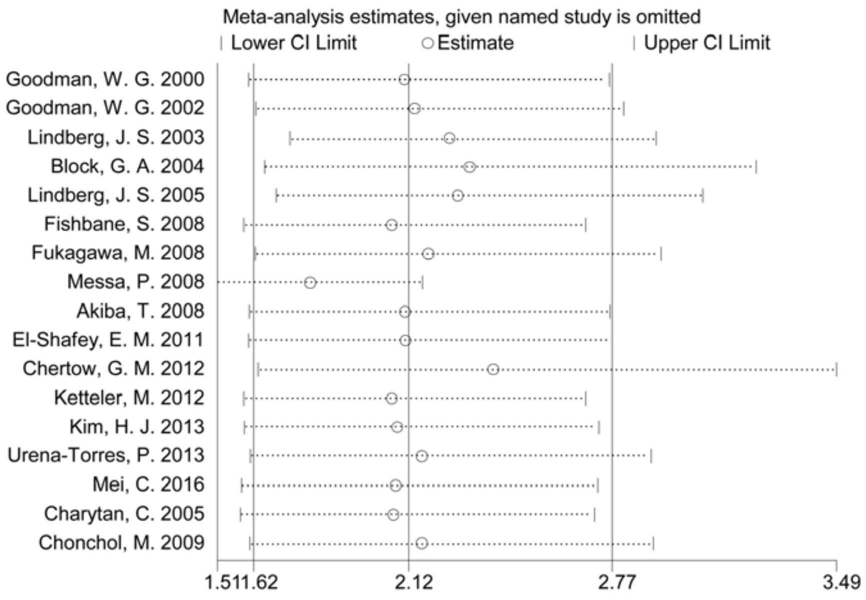

Figure S4. Sensitivity analysis of nausea in cinacalcet group versus control group. CI= confidence interval

Study or Subgroup	cinacalcet Mean	cinacalcet SD	cinacalcet Total	placebo or no treatment Mean	placebo or no treatment SD	placebo or no treatment Total	Weight	Std. Mean Difference IV, Random, 95% CI	Year
**5.3.1 treated with dialysis**									
Malluche, H. H. 2008	20.8	12.64	19	80.3	114.4	13	13.6%	-0.79 [-1.53, -0.06]	2008
Akiba, T. 2008	36.55	17.62	24	33.23	31.8	30	20.9%	0.12 [-0.41, 0.66]	2008
Fukagawa, M. 2008	46.6	51.98	72	43.86	25.95	71	34.3%	0.07 [-0.26, 0.39]	2008
**Subtotal (95% CI)**			**115**			**114**	**68.8%**	**-0.12 [-0.57, 0.34]**	
Heterogeneity: Tau² = 0.10; Chi² = 4.79, df = 2 (P = 0.09); I² = 58%									
Test for overall effect: Z = 0.49 (P = 0.62)									
**5.3.2 treated with renal transplant**									
Evenepoel, P. 2014	26.3	13.68	57	23.85	13.47	57	31.2%	0.18 [-0.19, 0.55]	2014
**Subtotal (95% CI)**			**57**			**57**	**31.2%**	**0.18 [-0.19, 0.55]**	
Heterogeneity: Not applicable									
Test for overall effect: Z = 0.96 (P = 0.34)									
**Total (95% CI)**			**172**			**171**	**100.0%**	**-0.00 [-0.31, 0.31]**	
Heterogeneity: Tau² = 0.05; Chi² = 5.61, df = 3 (P = 0.13); I² = 47%									
Test for overall effect: Z = 0.02 (P = 0.98)									
Test for subgroup differences: Chi² = 0.96, df = 1 (P = 0.33), I² = 0%									

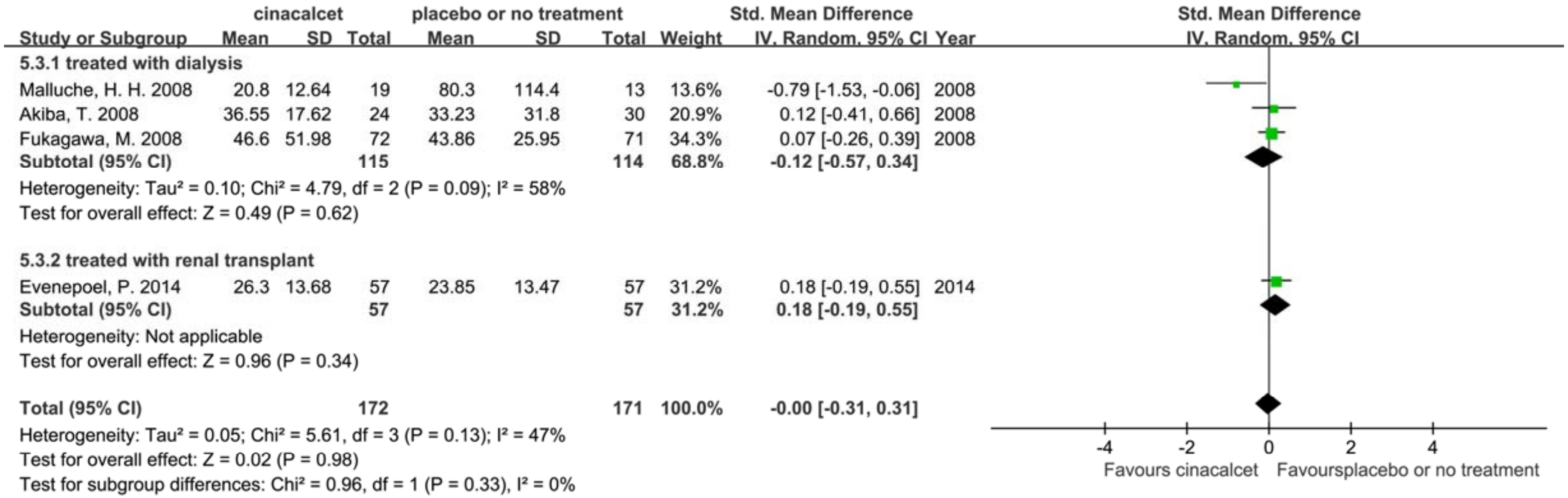

Figure S5. Forest plots for the level of BALP in cinacalcet group compared with control group. SD=Standard deviation; CI= confidence interval

	cinacalcet			placebo or no treatment				Std. Mean Difference	
Study or Subgroup	Mean	SD	Total	Mean	SD	Total	Weight	IV, Random, 95% CI	Year
**5.1.1 treated with dialysis**									
Akiba, T. 2008	129.59	66.4	24	164	88.95	30	30.2%	-0.43 [-0.97, 0.12]	2008
Fukagawa, M. 2008	107.9	66.9	72	169.5	90.7	71	35.2%	-0.77 [-1.11, -0.43]	2008
**Subtotal (95% CI)**			**96**			**101**	**65.4%**	**-0.67 [-0.98, -0.35]**	
Heterogeneity: Tau² = 0.01; Chi² = 1.11, df = 1 (P = 0.29); I² = 10%									
Test for overall effect: Z = 4.20 (P < 0.0001)									
**5.1.2 treated with renal transplant**									
Evenepoel, P. 2014	69.43	37.22	57	62.85	36.31	57	34.6%	0.18 [-0.19, 0.55]	2014
**Subtotal (95% CI)**			**57**			**57**	**34.6%**	**0.18 [-0.19, 0.55]**	
Heterogeneity: Not applicable									
Test for overall effect: Z = 0.95 (P = 0.34)									
**Total (95% CI)**			**153**			**158**	**100.0%**	**-0.34 [-0.96, 0.28]**	
Heterogeneity: Tau² = 0.25; Chi² = 13.83, df = 2 (P = 0.0010); I² = 86%									
Test for overall effect: Z = 1.07 (P = 0.28)									
Test for subgroup differences: Chi² = 11.78, df = 1 (P = 0.0006), I² = 91.5%									

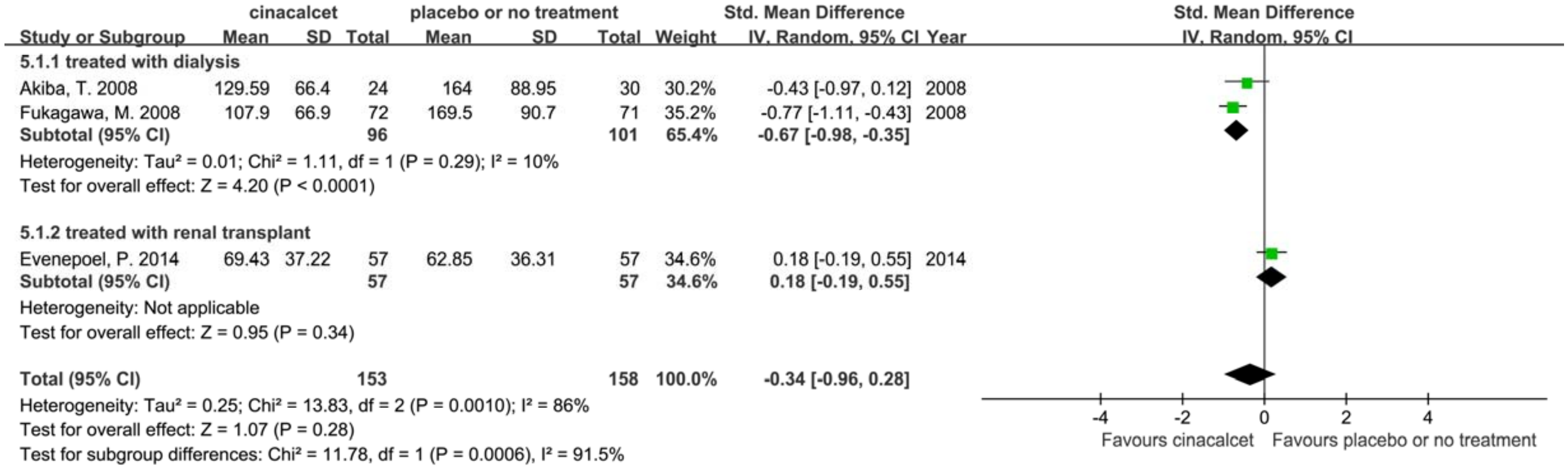

Figure S6. Forest plots for the level of osteocalcin in cinacalcet group compared with control group. SD=Standard deviation; CI= confidence interval

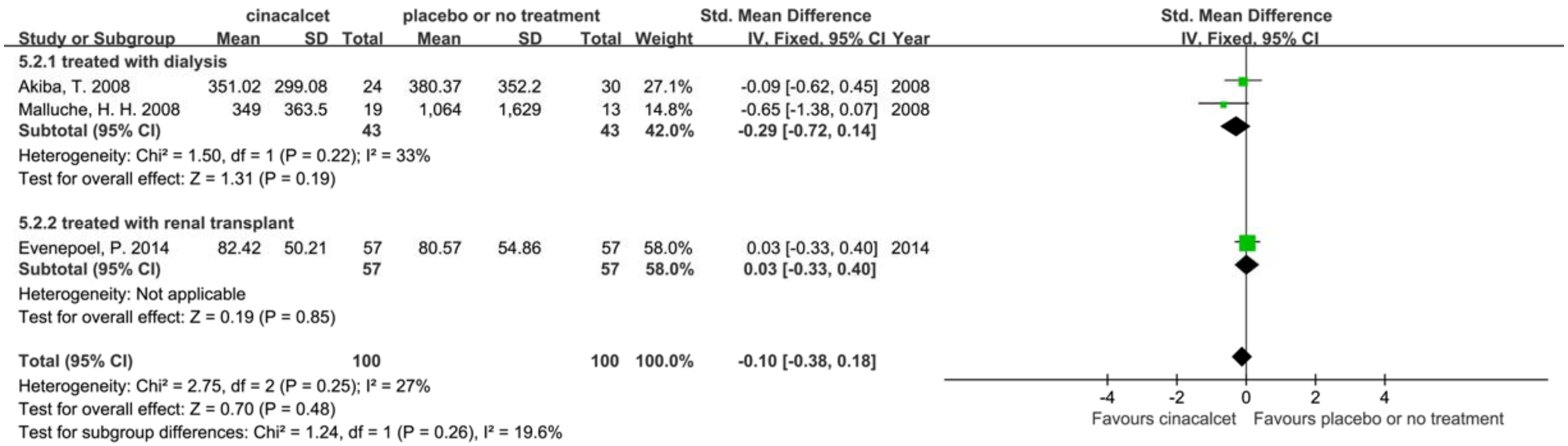

Figure S7. Forest plots for the level of urine NTx in cinacalcet group compared with control group. SD=Standard deviation; CI= confidence interval

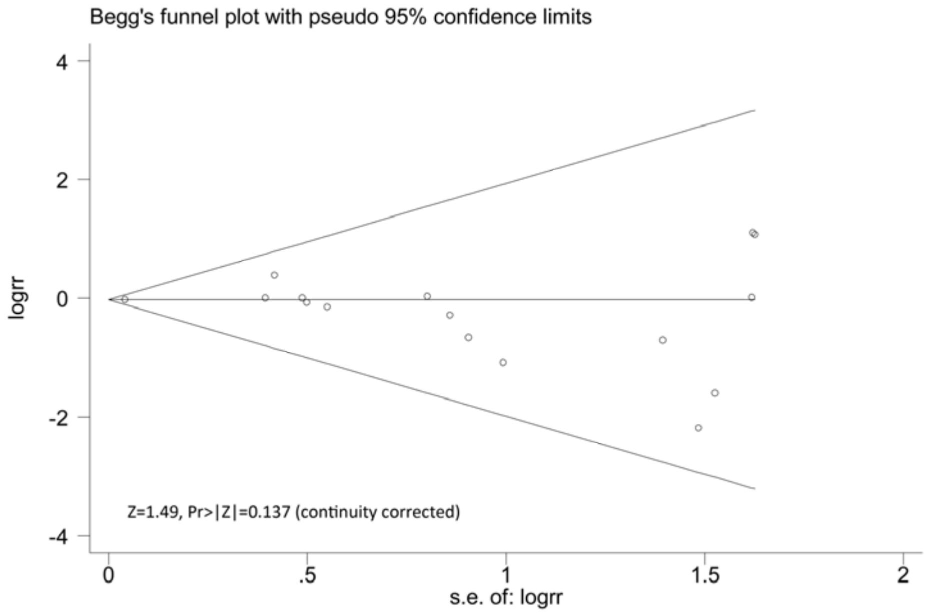

Figure S8. begg's funnel plot of all-cause mortality

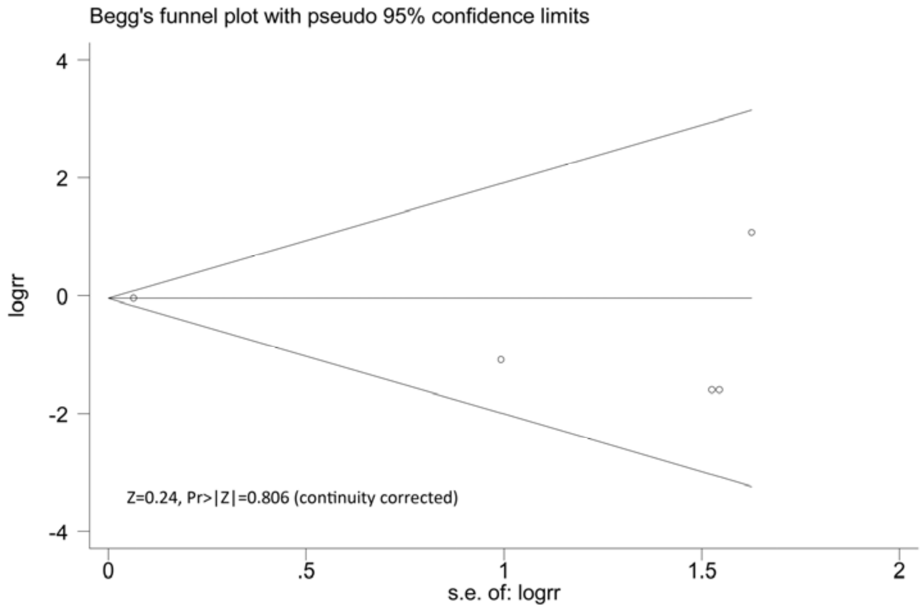

Figure S9. Begg’s funnel plot of cardiovascular mortality.

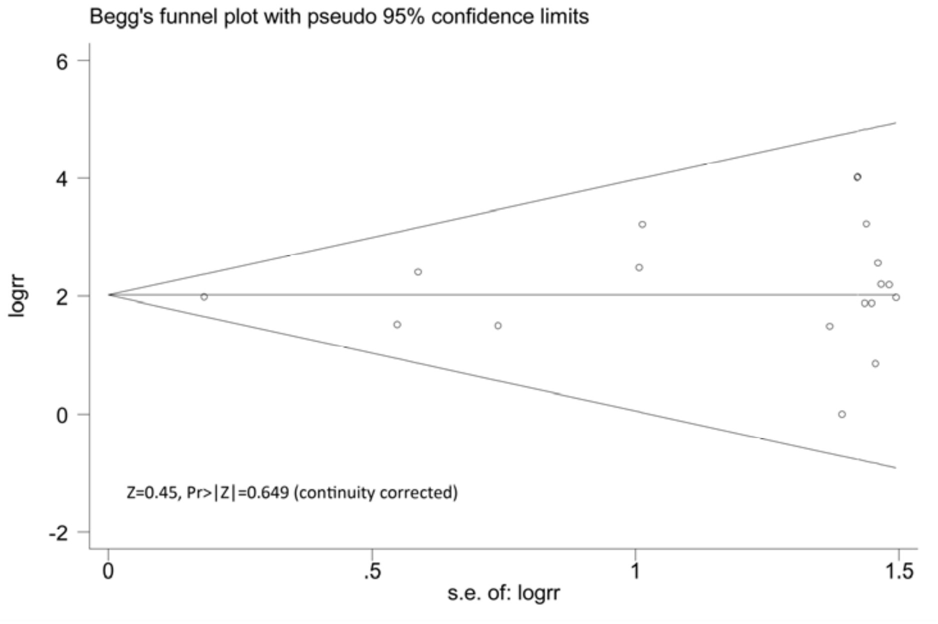

Figure S10. Begg’s funnel plot of hypocalcaemia.

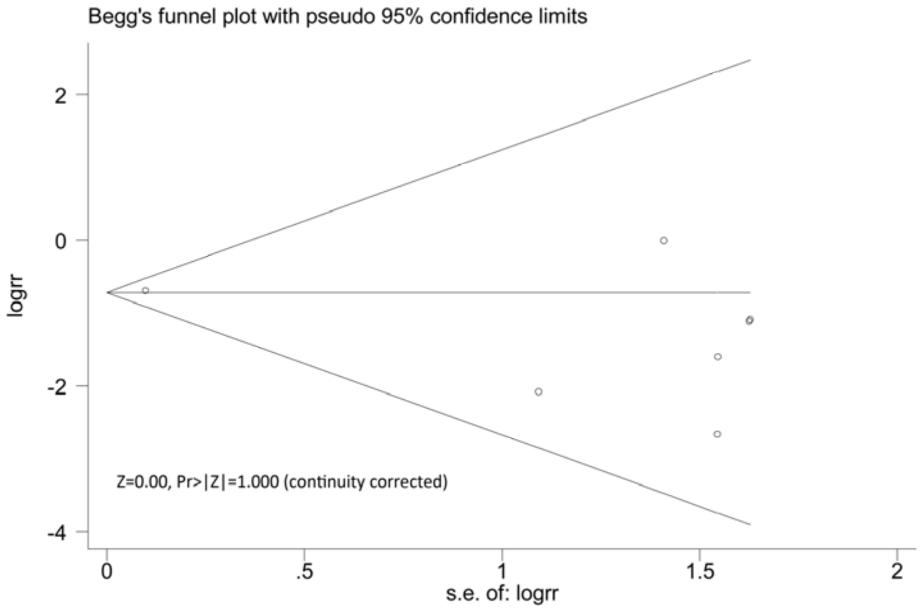

Figure S11. Begg's funnel plot of parathyroidectomy.

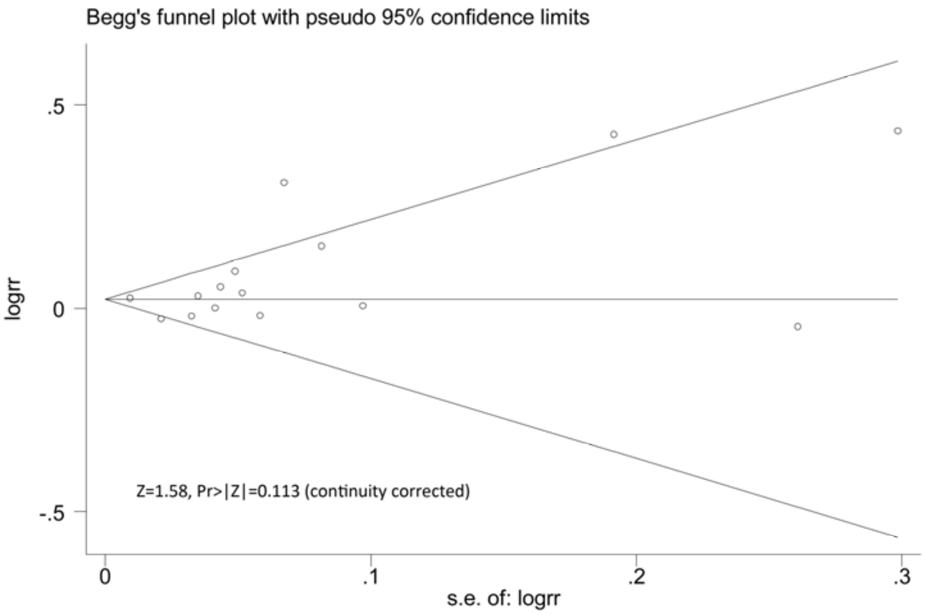

Figure S12. Begg’s funnel plot of all advert events.

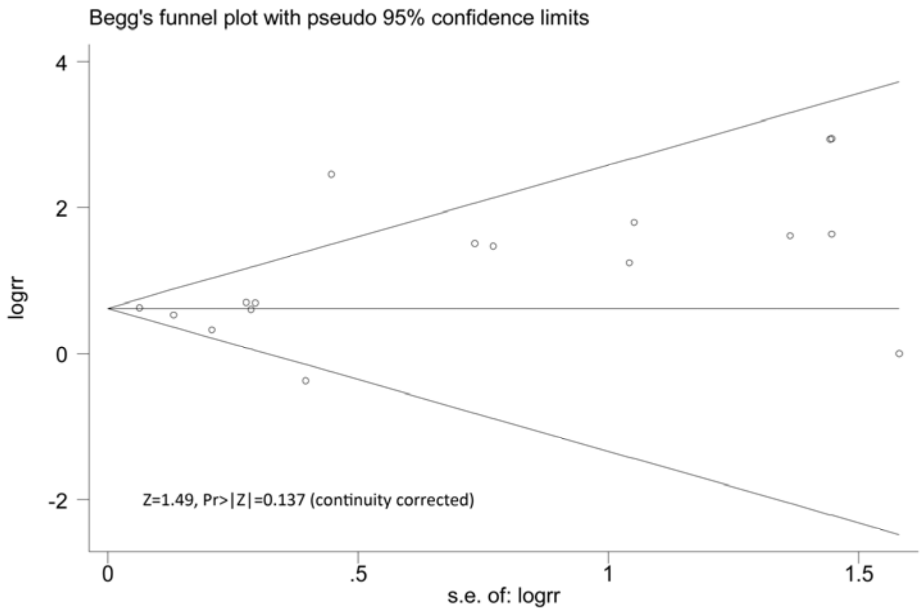

Figure 13. Begg's funnel plot of nausea.

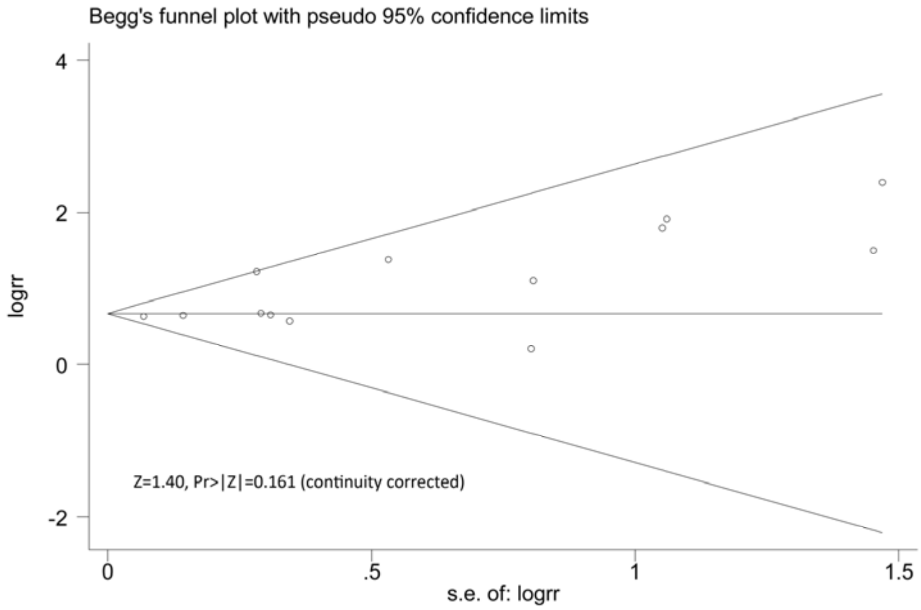

Figure S14. Begg's funnel plot of vomiting.

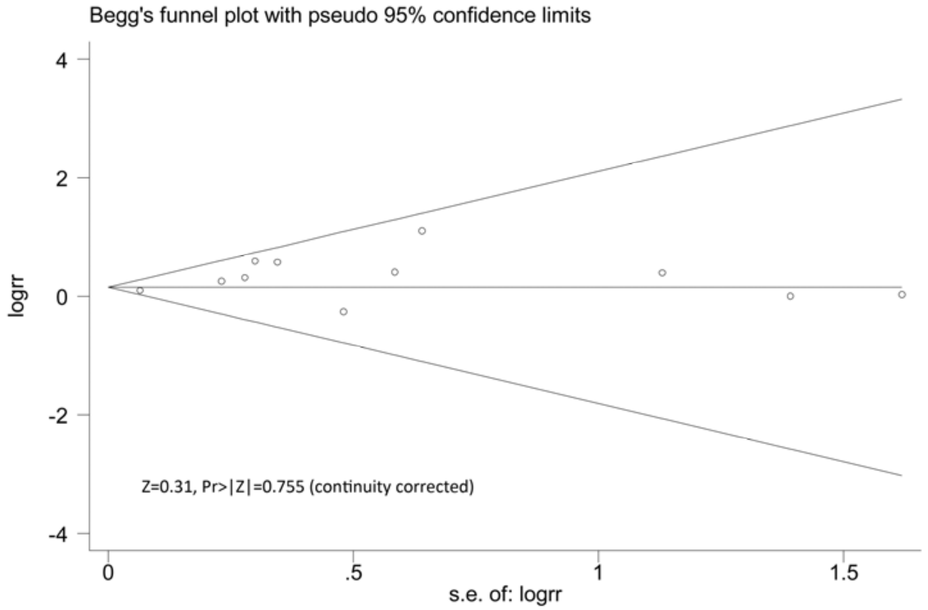

Figure S15. Begg’s funnel plot of diarrhea.
